# Significance of cuproptosis- related genes in the diagnosis and classification of psoriasis

**DOI:** 10.3389/fmolb.2023.1115091

**Published:** 2023-04-07

**Authors:** Qingyuan Lin, Jinchao Zhu, Jun Chen, Shouqiang Jia, Shengdong Nie

**Affiliations:** ^1^ School of Health Science and Engineering, University of Shanghai for Science and Technology, Shanghai, China; ^2^ Department of Pathology, Ninth People’s Hospital, Shanghai Jiao Tong University School of Medicine, Shanghai, China; ^3^ Department of Imaging, Jinan People’s Hospital Affiliated to Shandong First Medical University, Jinan, Shandong, China

**Keywords:** psoriasis, cuproptosis, diagnostic markers, drug sensitivity analysis, principal component analysis

## Abstract

Cuproptosis is a novel form of cell death linked to mitochondrial metabolism and is mediated by protein lipoylation. The mechanism of cuproptosis in many diseases, such as psoriasis, remains unclear. In this study, signature diagnostic markers of cuproptosis were screened by differential analysis between psoriatic and non-psoriatic patients. The differentially expressed cuproptosis-related genes (CRGs) for patients with psoriasis were screened using the GSE178197 dataset from the gene expression omnibus database. The biological roles of CRGs were identified by GO and KEGG enrichment analyses, and the candidates of cuproptosis-related regulators were selected from a nomogram model. The consensus clustering approach was used to classify psoriasis into clusters and the principal component analysis algorithms were constructed to calculate the cuproptosis score. Finally, latent diagnostic markers and drug sensitivity were analyzed using the pRRophetic R package. The differential analysis revealed that CRGs (MTF1, ATP7B, and SLC31A1) are significantly expressed in psoriatic patients. GO and KEGG enrichment analyses showed that the biological functions of CRGs were mainly related to acetyl-CoA metabolic processes, the mitochondrial matrix, and acyltransferase activity. Compared to the machine learning method used, the random forest model has higher accuracy in the occurrence of cuproptosis. However, the decision curve of the candidate cuproptosis regulators analysis showed that patients can benefit from the nomogram model. The consensus clustering analysis showed that psoriasis can be grouped into three patterns of cuproptosis (clusterA, clusterB, and clusterC) based on selected important regulators of cuproptosis. In advance, we analyzed the immune characteristics of patients and found that clusterA was associated with T cells, clusterB with neutrophil cells, and clusterC predominantly with B cells. Drug sensitivity analysis showed that three cuproptosis regulators (ATP7B, SLC31A1, and MTF1) were associated with the drug sensitivity. This study provides insight into the specific biological functions and related mechanisms of CRGs in the development of psoriasis and indicates that cuproptosis plays a non-negligible role. These results may help guide future treatment strategies for psoriasis.

## Introduction

Psoriasis is a clinically common chronic, hereditary, systemic, immune-abnormal inflammatory disease (Kamiya et al., 2019), which is difficult to cure and easy to relapse. It is characterized by clear borders and white plaques on the skin. Psoriasis is the most common chronic inflammatory disease among skin diseases and its onset spans all age groups (Griffiths and Barker, 2007; Parisi et al., 2013). It has different clinical phenotypes, but the most frequent and most easily recognised is chronic plaque or psoriasis vulgaris (Griffiths et al., 2021). Symptoms of psoriasis can affect other organs and include itching or burning (Michalek et al., 2017; Feldman, 2020). Although psoriasis is primarily hereditary, it can be induced by air pollution (Puri et al., 2017), drugs (Kamiya et al., 2019), and vaccinations (Rahier et al., 2010) and alcohol (Brenaut et al., 2013), but studies have shown that no causality between alcohol consumption and psoriasis (Chang et al., 2022). The pathogenesis of psoriasis is still not fully understood. Therefore, exploring the specific mechanism of psoriasis has crucial scientific value and clinical significance.

Previous studies have shown that trace elements play essential roles in skin metabolism, such as keratinization and melanin formation, as well as immune and inflammatory responses (Aggarwal et al., 2021). Cadmium is one of the main factors affecting the pathogenesis of psoriasis, where patients with severe cases were found to have higher blood cadmium levels (Liaw et al., 2017). Another study showed that serum copper levels were significantly higher in patients with psoriasis compared to those without (Lei et al., 2019). Abnormal levels of serum copper and zinc are essential for the development of skin diseases, including the pathophysiological process of psoriasis (Basavaraj et al., 2009). A recent study showed that copper induces cell death *via* the tricarboxylic acid cycle, providing strong evidence for cuproptosis (Tsvetkov et al., 2022). However, the molecular mechanisms involved in this process are unclear.

Psoriasis is an immunomodulatory polygenic disease (Lei et al., 2019). The importance of T cells in its pathogenesis has been known since the early 1980s when it was confirmed by clinical trials using cyclosporine (Ellis et al., 1986; Griffiths et al., 1986). T helper 17 (Th17) signaling plays a pivotal role in the immune mechanisms of psoriasis (Shimizu et al., 2019). Infection can upregulate T helper 17 (Th17) signaling and exacerbate psoriasis (Yu et al., 2022). In addition, cytokine-inducible SH2-containing protein 1 (CIS1) induced by Th2 cytokines has the ability to change the response of epidermal keratinocytes to IL-17A by suppression of Src family kinases (Tohyama et al., 2021). Although current studies suggest that the accumulation of copper in serum can induce psoriasis, its specific mechanism and the genes involved have not been elucidated. In this study, we comprehensively assessed the role of cuproptosis in the diagnosis and subtype classification of psoriasis based on the GSE178197 dataset from the Gene Expression Omnibus (GEO) database. We established a genetic model for predicting psoriasis susceptibility based on three candidate cuproptosis-related genes (CRGs), MTF1, ATP7B, and SLC31A1, and found that patients can gain clinical benefits based on this model. Furthermore, we revealed three distinct modes of cuproptosis that are significantly associated with T cells, neutrophils, and B cells. It was shown that the cuproptosis pattern may affect the development of psoriasis and immune system regulation.

## Materials and methods

### Data collection and pre-processing

The GSE178197 dataset from the GEO database was used for data processing. It contained expression data for 15 healthy patients and 69 patients with psoriasis. We used the inclusion and exclusion criteria of recruitment as follow: the patients histological features including epidermal hyperplasia; dilated, prominent blood vessels in the dermis; and an inflammatory infiltrate of leucocytes, predominantly into the dermis, the hyperplastic epidermal changes are associated with an under expression of markers of keratinocyte differentiation, including keratins K1 and K10; loss of the granular cell layer; parakeratosis; elongation of rete ridges; and the presence of micropustules of Kogoj and microabscesses of Munro (Griffiths and Barker, 2007). The patients histological features including: Histology of uninvolved, clinically symptomless areas of skin is normal. Differential analysis of 19 CRGs (NFE2L2, NLRP3, ATP7B, ATP7A, SLC31A1, FDX1, LIAS, LIPT1, LIPT2, DLD, DLAT, PDHA1, PDHB, MTF1, GLS, CDKN2A, DBT, GCSH, DLST) between psoriatic and non-psoriatic patients was performed to identify the significantly expressed genes (Tsvetkov et al., 2022; Zhang et al., 2022). These genes were then selected as key CRGs.

### CRGs correlation analysis

To check the correlation between copper death genes in psoriasis, the study carried out the correlation between copper death genes. The filtering standard of correlation coefficient is greater than or equal to 0.3, and the filtering standard of correlation test *p*-value is less than or equal to 0.05. Use the “ggplot2,”, “ggpubr” and “ggExtra” packages in R to visualize the correlation between copper death genes.

### Construction of random forest model and support vector machine models

Random forest (RF) and support vector machine (SVM) models were used as training models to predict the presence of psoriasis. The R programming packages used to process the inverse cumulative distribution and B-curve of residuals, as well as the receiver work characteristic (ROC) curve, included “caret,” “DALEX,” “ggplot2,” “randomForest,” “kernlab,” and “pROC.” The “randomForest” package was also used to build the RF model. We selected 19 known cuproptosis genes to predict the presence of psoriasis in patients, analyzed their importance, and finally screened for the most important genes. Machine learning with separation margin (vector) maximization (support), known as SVM learning, is a powerful classification tool that has been used for cancer genome or subtype classification (Huang et al., 2018). Here, we used the SVM algorithm to find an optimal hyperplane that can distinguish psoriasis and non-psoriasis well. Additionally, we plotted ROC curves to predict the accuracy of the model and signature gene.

### Construction of a nomogram model

Nomograms can be used for multi-index combined diagnosis and the prediction of disease incidence or progression, and have increasingly been used in clinical cases (Lei et al., 2016). Therefore, we constructed a nomogram model using the “rms” and “rmda” R packages to predict the incidence of psoriasis based on the selected candidate cuproptosis genes. A calibration curve was used to evaluate the agreement of our predicted values with reality, where the abscissa represents the predicted probability and the ordinate represents the actual probability. Decision curve analysis of candidate cuproptosis genes was performed and clinical impact curves were drawn to assess whether decisions based on the cuproptosis model would benefit patients.

### Molecular subtyping and estimation of cuproptosis-related gene signaling

Consensus clustering, an unsupervised clustering method commonly used in cancer subtype classification, can distinguish samples into several subtypes according to different omics data sets. Consensus clustering is also appropriate for discovering new disease subtypes. Based on significant cuproptosis genes, a consensus clustering method was performed using the “ConensusClusterPlus” R package. Using this method, distinct cuproptosis patterns can be identified and optimal k values selected (Wilkerson and Hayes, 2010). To quantify cuproptosis patterns in our study, a principal component analysis (PCA) algorithm was used to calculate cuproptosis gene scores for each sample.

### Estimation of immune cell infiltration

Single sample gene set enrichment analysis (ssGSEA), an extension of the GSEA method, was used for a single sample that could not undergo GSEA. This study used ssGSEA to evaluate the abundance of immune cells in patients with psoriasis. Gene expression levels of the samples were sequenced using ssGSEA and then collated. The results were used to obtain the abundance level of immune cells in each sample. In addition, we further analyzed the relationship between each candidate cuproptosis gene and immune cell abundance.

### Drug sensitivity analysis

With the continuous advancement of precision medicine, people’s demand for personalized treatment has increased. The sensitivity of genes and drugs is a significant factor in personalized treatment. Robust prediction of *in vivo* chemotherapy response by collecting pretreatment (baseline) gene expression and drug sensitivity data from cancer cell lines has been a long-standing, controversial issue in pharmacogenomics. Based on this, Paul Geeleher et al. proposed a method to solve the problem (Geeleher et al., 2014). The “pRRophetic” package works by building statistical models from gene expression and drug sensitivity data from a vast set of cancer cell lines and then applying these models to gene expression data from primary tumor biopsies (Geeleher et al., 2014). This study used “pRRophetic” to calculate the sensitivity between candidate cuproptosis genes and various drugs.

### Immunohistochemistry analysis of clinical samples

Immunohistochemical analysis of paraffin-embedded psoriasis tissues with the following rabbit monoclonal antibodies: anti-ATP7B (ER62773, dilution 1:300; HUABIO), anti-SLC31A1 (67221-1-lg, dilution 1:1000; ProteinTech), and anti-MTF1 (HA500296, dilution 1:200; HUABIO). In this study, the paraffin-embedded psoriasis tissues were surgically removed from the patients at the dermatology department and presented to the pathology department for pathological diagnosis of psoriasis with the diagnosis of psoriasis, and the paraffin-embedded psoriasis tissues were kept in the pathology department, the study was approved and used the paraffin-embedded psoriasis tissues by the ethics committee of the Ninth People’s Hospital affiliated to Shanghai Jiao Tong University School of Medicine (Approval No: SH9H-2023-146-1). Tests were performed using a BenchMark automatic immunohistochemical instrument. Positive and negative controls were prepared according to the manufacturer’s recommendations. Immunoreactivity results were recorded as negative without epidermal insufficiency or hyperkeratosis cell expression as positive.

### Statistical analysis

All statistics were performed with R 4.1.2 software. Linear regression analysis was used to explore the correlation between cuproptosis genes. The RF and SVM models were used to screen cuproptosis candidate genes. The Wilcoxon test was used to examine the differential expression of genes between psoriasis and non-psoriasis, and Spearman’s rank correlation test was used to evaluate the significance of the correlation. Statistical significance was set as *p* < 0.05.

## Results

### Differential expression of cuproptosis-related genes in psoriasis

**FIGURE 1:**
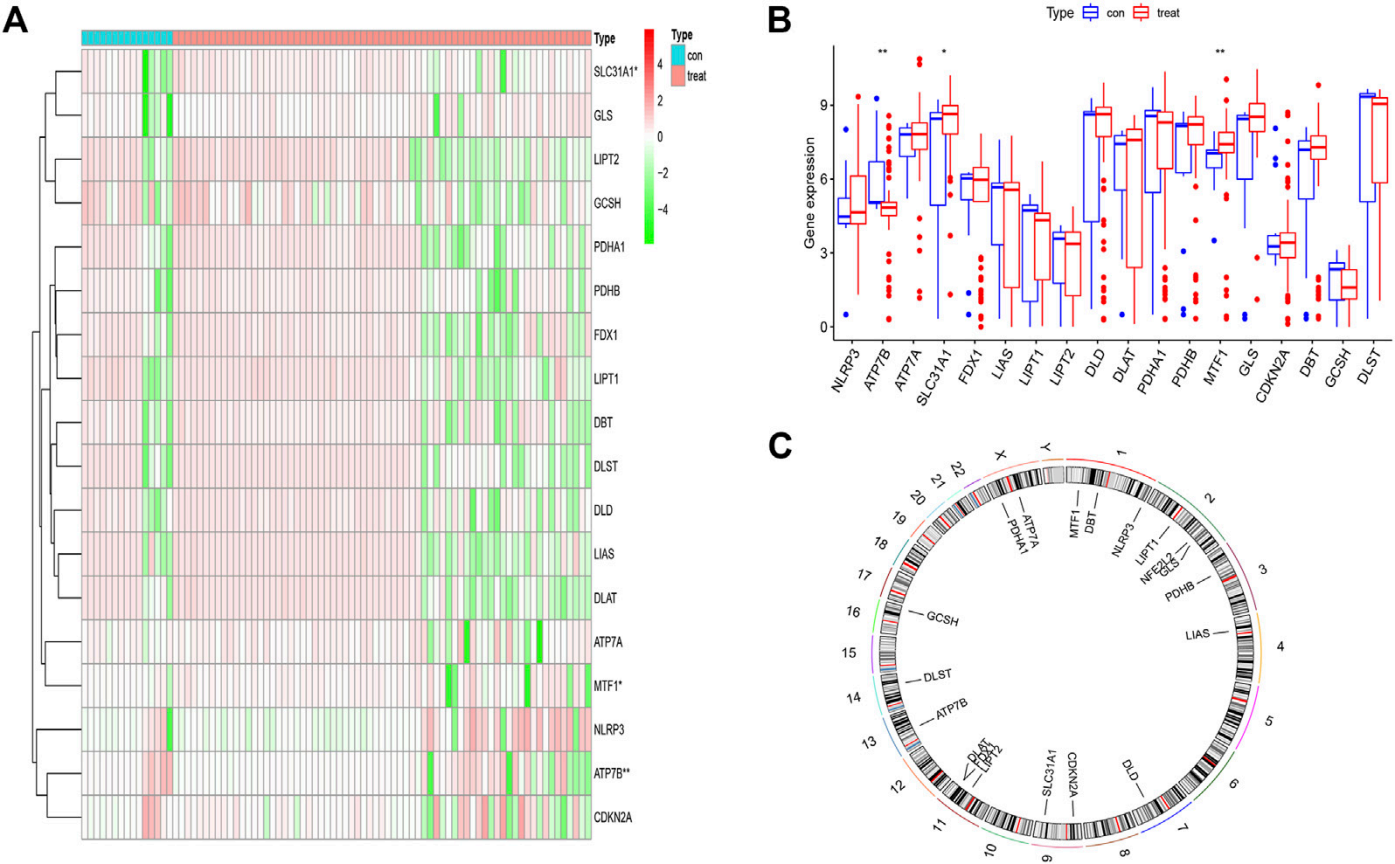
The landscape of the cuproptosis-related genes in psoriasis. **(A,B)** Expression heat map of the 21 m6A-related regulators in non-psoriasis and psoriasis patients. **(C)** Chromosomal positions of the 19 cuproptosis-related genes. *p < 0.05, **p < 0.01, and ***p < 0.001.

We obtained the differential expression levels of 19 CRGs between the psoriasis and non-psoriasis datasets using the “limma” R package and visualized them using “pheatmap” and “ggpubr” R packages. Comparing the differentially expressed genes, three CRGs were significantly expressed; namely, MTF1, ATP7B, and SLC31A1. We found that the expression of ATP7B was lower in psoriatic patients compared to non-psoriatic patients, whereas MTF1 and SLC31A1 were overexpressed (Figures 1A, B). We also determined the chromosomal location of the 19 cuproptosis genes and visualized them using the “RCiros” R package (Figure 1C).

### The correlation and protein interaction analysis of cuproptosis-related genes

**FIGURE 2:**
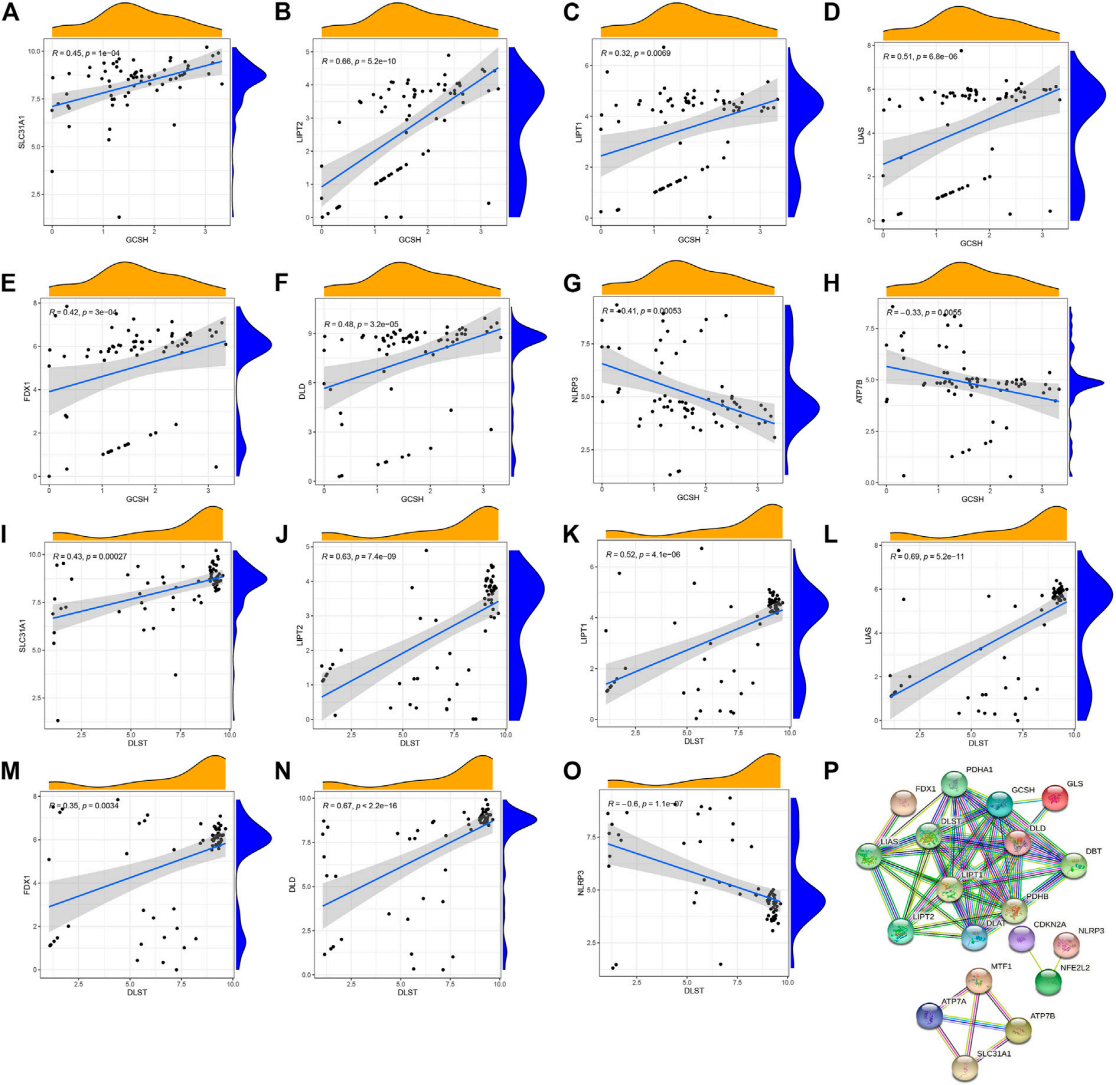
Correlation between writers and erasers in psoriasis. **(A–F)** The expression levels of SLC31A1, LIPT2, LIPT1, LIAS, FDX1 and DLD were highly positively correlated with GCSH. **(G,H)** The expression levels of NLRP3 and ATP7B were negatively correlated with GCSH. **(I–N)** The expression levels of SLC31A1, LIPT2, LIPT1, LIAS, FDX1 and DLD were highly positively correlated with DLST. **(O)** NLRP3 expression level was negatively correlated with DLST. **(P)** The protein interactions of 19 cuproptosis-related genes.

To explore the correlation between cuproptosis genes in patients with psoriasis, we used linear regression analysis and found that the expression levels of FDX1, SLC31A1, DLD, LIAS, LIPT2, and LIPT1 were highly positively correlated with GCSH (Figures 2A–F), whereas the expression levels of ATP7B and NLRP3 showed a negative correlation (Figures 2G, H). In addition, the expression levels of LIAS, SLC31A1, LIPT2, LIPT1, FDX1, and DLD were highly positively correlated with DLST (Figures 2I–N), whereas the expression levels of NLRP3 showed a negative correlation (Figure 2O). Using the STRING database (https://string-db.org) to analyze the protein interaction network relationship, we found that the 19 cuproptosis genes are co-expressed. Co-expression of ATP7B, SLC31A1, and MTF1 suggests that these key cuproptosis genes may jointly influence psoriasis development (Figure 2P).

### Construction of the prognostic signature of cuproptosis-related genes in psoriasis

**FIGURE 3:**
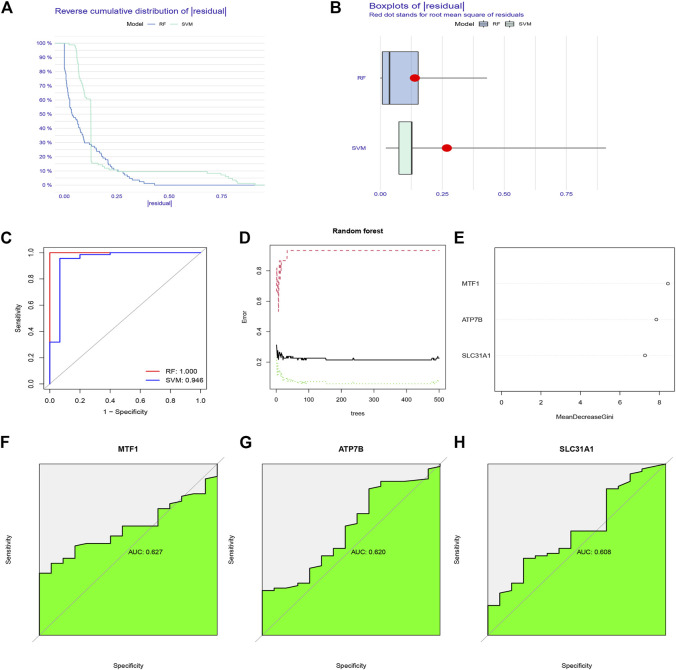
The construction of Random forest (RF) model for cuproptosis regulators in psoriasis. **(A)** Reverse cumulative distribution of residual was plotted to show the residual distribution of RF and support vector machine (SVM) model. **(B)** Boxplots of residual was plotted to show the residual distribution of RF and SVM model. **(C)** The AUC value of the ROC curve shows that the RF model has higher accuracy than the SVM model. **(D)** Ten-fold cross-validation curve indicated that the RF model has the highest accuracy. **(E)** The importance of the 19 cuproptosis regulators based on the RF model. **(F–H)** ROC curve prediction of MTF1, ATP7B, and SLC31A1, indicating that the three model genes can well predict the occurrence of psoriasis.

To select prognostic genes from the 19 CRGs, we constructed RF and SVM models to predict the presence of psoriasis. Both inverse cumulative distribution (Figure 3A) and box plot of residuals (Figure 3B) showed that the RF model had the smallest residuals, indicating that it has a higher accuracy for predicting the presence of psoriasis. Moreover, we also evaluated the model using an ROC curve. The AUC value of the ROC curve also showed that the RF model has higher accuracy than the SVM model (Figure 3C). On this basis, we performed a tenfold cross-validation, showing that the RF model had the highest accuracy (Figure 3D), we sorted 84 samples randomly and ran 999 permutation testing (Supplementary Figure S1), and then visualized essential candidate genes (Figure 3E). The ROC curve prediction of the explored model genes (MTF1, ATP7B, and SLC31A1) showed that they could be used to reasonably predict the presence of psoriasis (Figures 3F–H).

**FIGURE 4:**
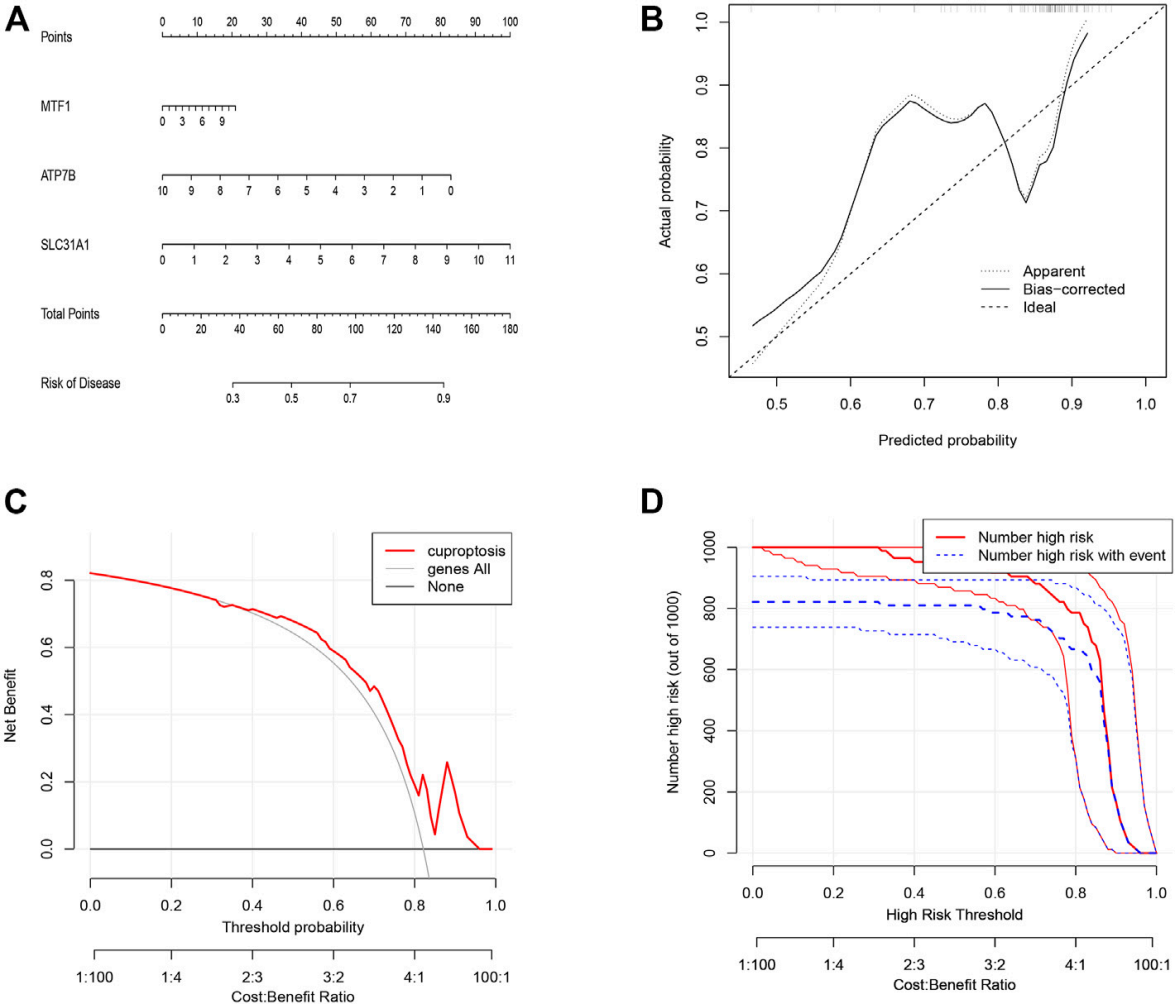
The construction of the nomogram model for cuproptosis regulators in psoriasis. **(A)** Construction of the nomogram model based on the three candidate cuproptosis regulators. **(B)** The calibration curve shows that the predictions of the nomogram model are accurate. **(C)** Nomogram-based decision-making may benefit psoriasis patients. **(D)** Assessing the clinical impact of the nomogram model by clinical impact curves.

To predict the presence of the three candidate cuproptosis genes in psoriasis, we constructed the cuproptosis gene nomogram model using “rms” and “rmda” in R (Figure 4A). The calibration (Figure 4B) and DCA curves showed that the predictiveness of the nomogram model is accurate and is an excellent predictor of the incidence of psoriasis (Figure 4C). In addition, the clinical impact curve revealed that the predictive power of the nomogram was significant (Figure 4D).

### Three distinct patterns of cuproptosis

**FIGURE 5:**
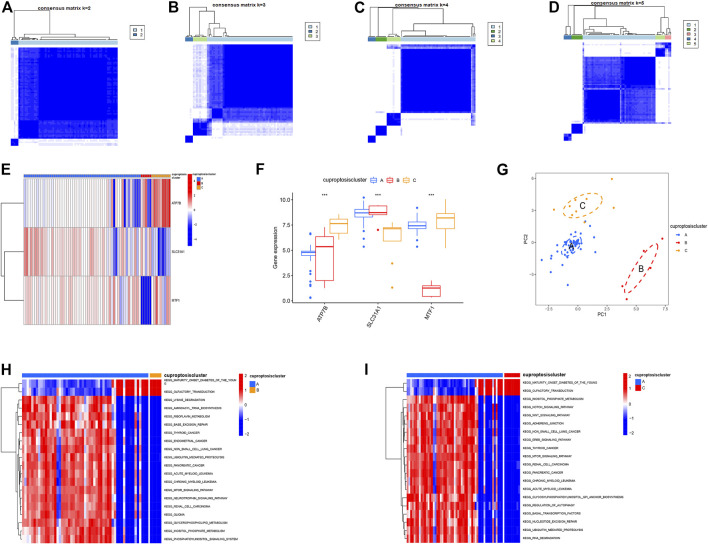
The consensus clustering analysis of cuproptosis regulators. **(A–D)** Consensus matrices of the three significant cuproptosis regulators for k = 2–5, **(E)** Expression heatmap of the three significant cuproptosis regulators in clusterA, clusterB and clusterC, **(F)** Differential expression histogram of the three significant cuproptosis regulators in clusterA, clusterB and clusterC. **(G)** Principal component analysis for the expression profiles of the three significant cuproptosis regulators. **(H,I)** GSVA showed that ClusterA was significantly enriched in oncogenic pathways, and clusterB and clusterC were mainly associated with diabetes and olfactory conduction in young adults.

Using the “ConensusClusterPlus” R package and co-clustering method, different cuproptosis patterns were identified based on the prognostic CRGs. Three cuproptosis patterns were identified (Figures 5A–D). ClusterA, clusterB, and clusterC contained 55, 5, and 9 cases, respectively (Supplementary Figure S1). Then, heatmaps and boxplots were drawn to illustrate the differential expression of the key cuproptosis genes in each cluster. The levels of MTF1 and ATP7B in clusterC were higher than those in clusterA and clusterB, while converse results were obtained for SLC31A1 (Figures 5E, F). The PCA showed that the significant cuproptosis genes could be completely distinguished in all cuproptosis patterns (Figure 5G). To explore the biological behavior between these different cuproptosis patterns, we performed GSVA enrichment analysis. ClusterA was significantly enriched in oncogenic pathways, including MTOR signaling, WNT signaling, and other oncogenic pathways. ClusterB and clusterC were mainly related to diabetes and olfactory conduction in young adults (Figures 5H, I).

**FIGURE 6:**
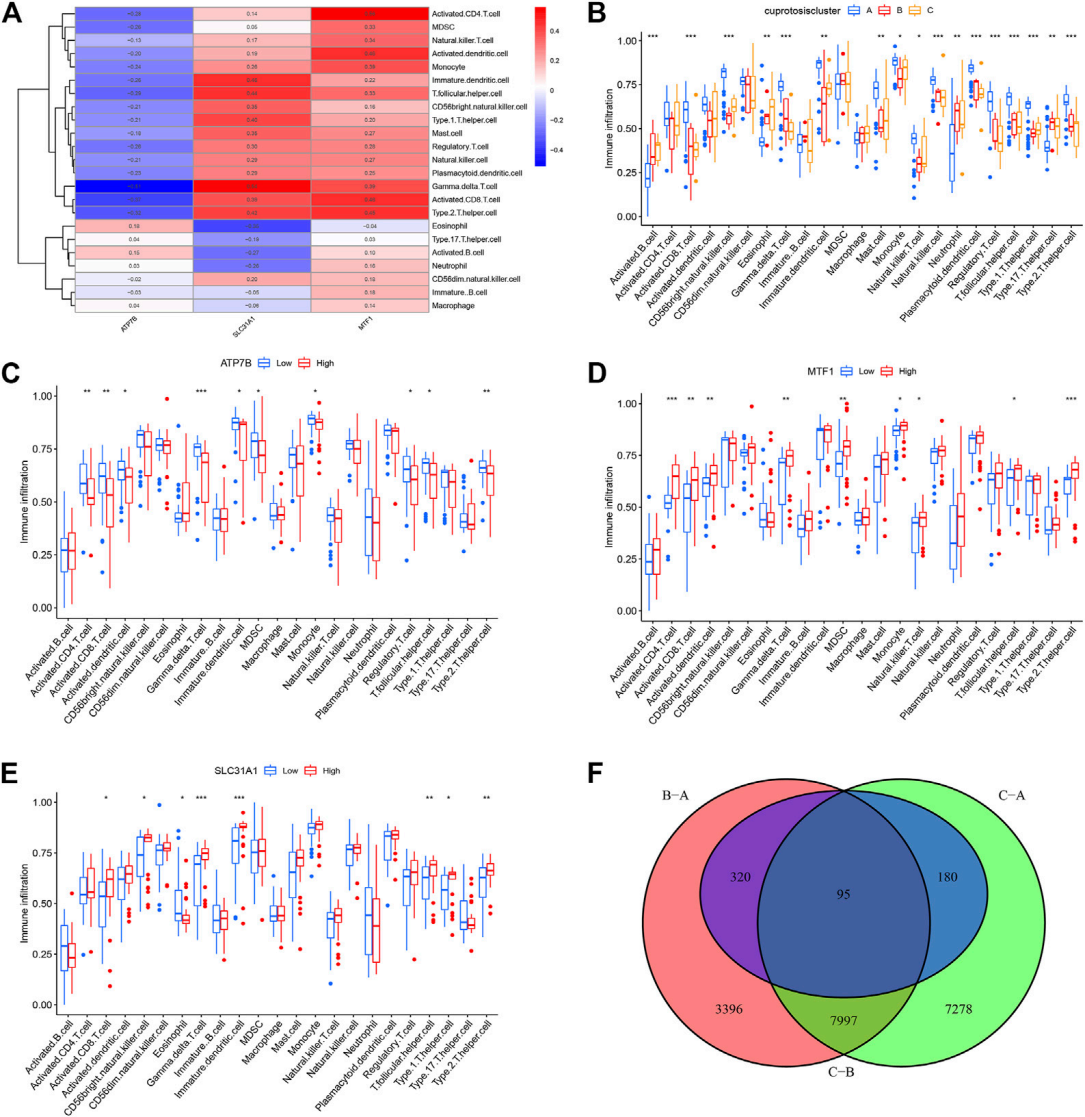
Single sample gene set enrichment analysis. **(A)** Correlation between infiltrating immune cells and the 3 significant cuproptosis regulators. **(B)** Differential immune cell infiltration between clusterA, clusterB and clusterC. **(C–E)** Difference in the abundance of infiltrating immune cells between high and low ATP7B, MTF1 and SCL31A1 expression groups. **(F)** The intersection of copper death differential genes in three copper death types. *p < 0.05, **p < 0.01, and ***p < 0.001.

Using Spearman’s correlation analysis in ssGSEA, we examined the correlation between cuproptosis genes and immune cells. We found that MTF1 and SLC31A1 were positively associated with most immune cells, whereas ATP7B was not (Figure 6A). Moreover, we analyzed the differences in immune cell infiltration among the three different cuproptosis patterns and discovered that they were significantly associated with most immune cells, indicating that immune infiltration plays a crucial role in the development of psoriasis (Figure 6B).

Based on this, we explored the correlation between immune cell infiltration and the three prognostic CRGs. In contrast to patients that showed high ATP7B expression, patients with low expression showed an increase in immune cell infiltration (Figure 6C). On the contrary, high expression of MTF1 and SLC31A1 could increase immune cell infiltration in patients (Figures 6D, E). Finally, we conducted a difference analysis between cuproptosis types. The logFC filter value was 0.5 and the adjusted *p*-value was 0.05. Intersections of genes with differences between different types are shown in Figure 6F. Furthermore, we cluster the gene correlation matrix and analyze the correlation between genes (Supplementary Figure S2).

### Identification and validation of cuproptosis patterns

**FIGURE 7:**
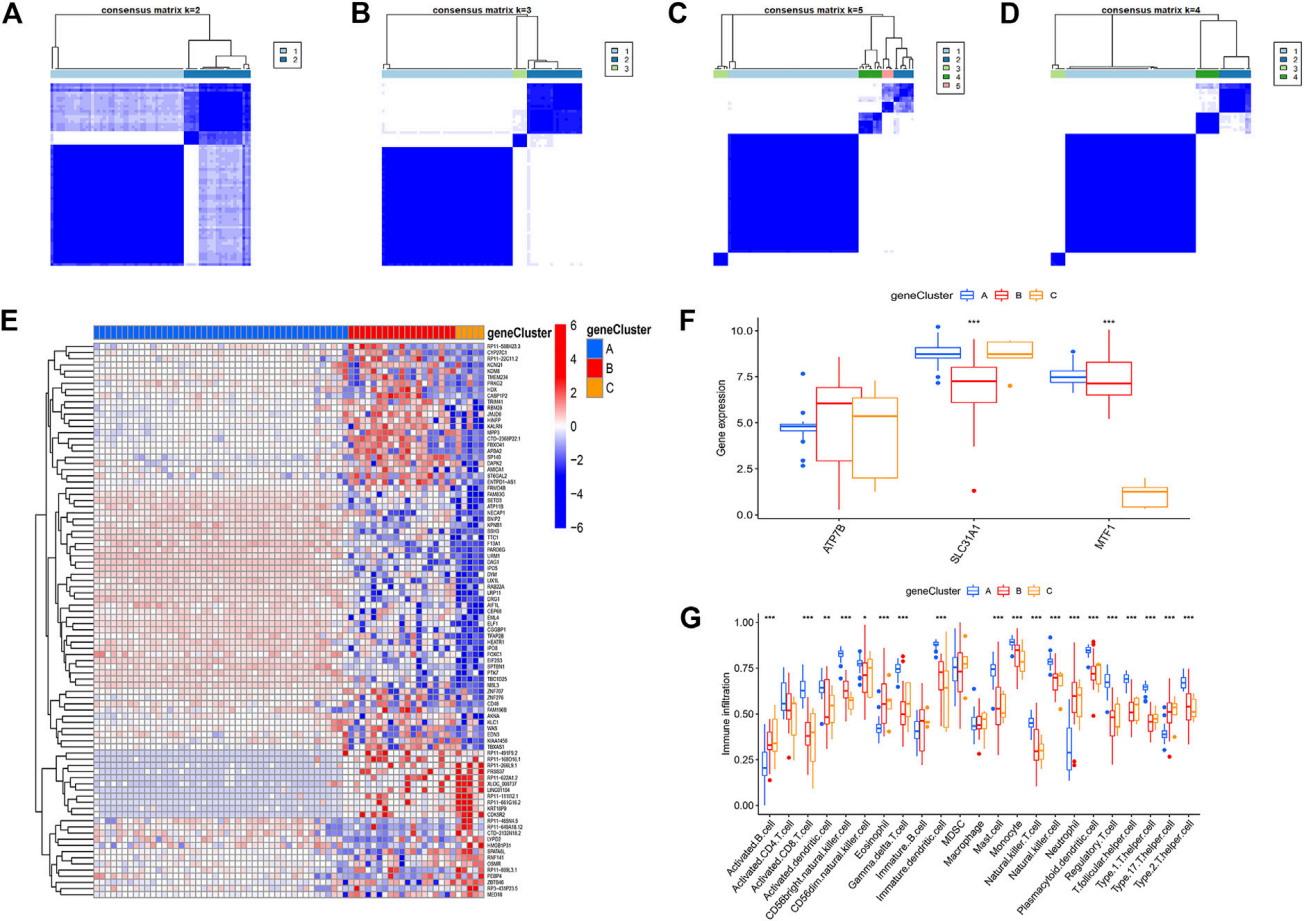
The consensus clustering of the 90 cuproptosis -related DEGs in childhood asthma. **(A–D)** Consensus matrices of the 90 cuproptosis-related DEGs for k = 2–5. **(E)** Expression heat map of the 119 cuproptosis-related DEGs in gene clusterA, gene clusterB and gene clusterC. **(F)** Differential expression histogram of the 3 significant cuproptosis regulators in gene clusterA, gene clusterB and gene clusterC. **(G)** Differential immune cell infiltration between gene clusterA, gene clusterB and gene clusterC.

To further identify the patterns of cuproptosis, we conducted consensus clustering analysis. When the psoriasis patients were grouped into different genomic subtypes, we found three distinct patterns of cuproptosis genes (clusterA, clusterB, and clusterC) that are consistent with the grouping of cuproptosis patterns (Figures 7A–D). The expression levels of cuproptosis-related differential genes in clusterA, clusterB, and clusterC are shown in Figures 7E–G. The results showed that the expression levels of the core cuproptosis genes and the infiltration of immune cells between gene clusterA, gene clusterB, and gene clusterC had the same cuproptosis pattern.

**FIGURE 8:**
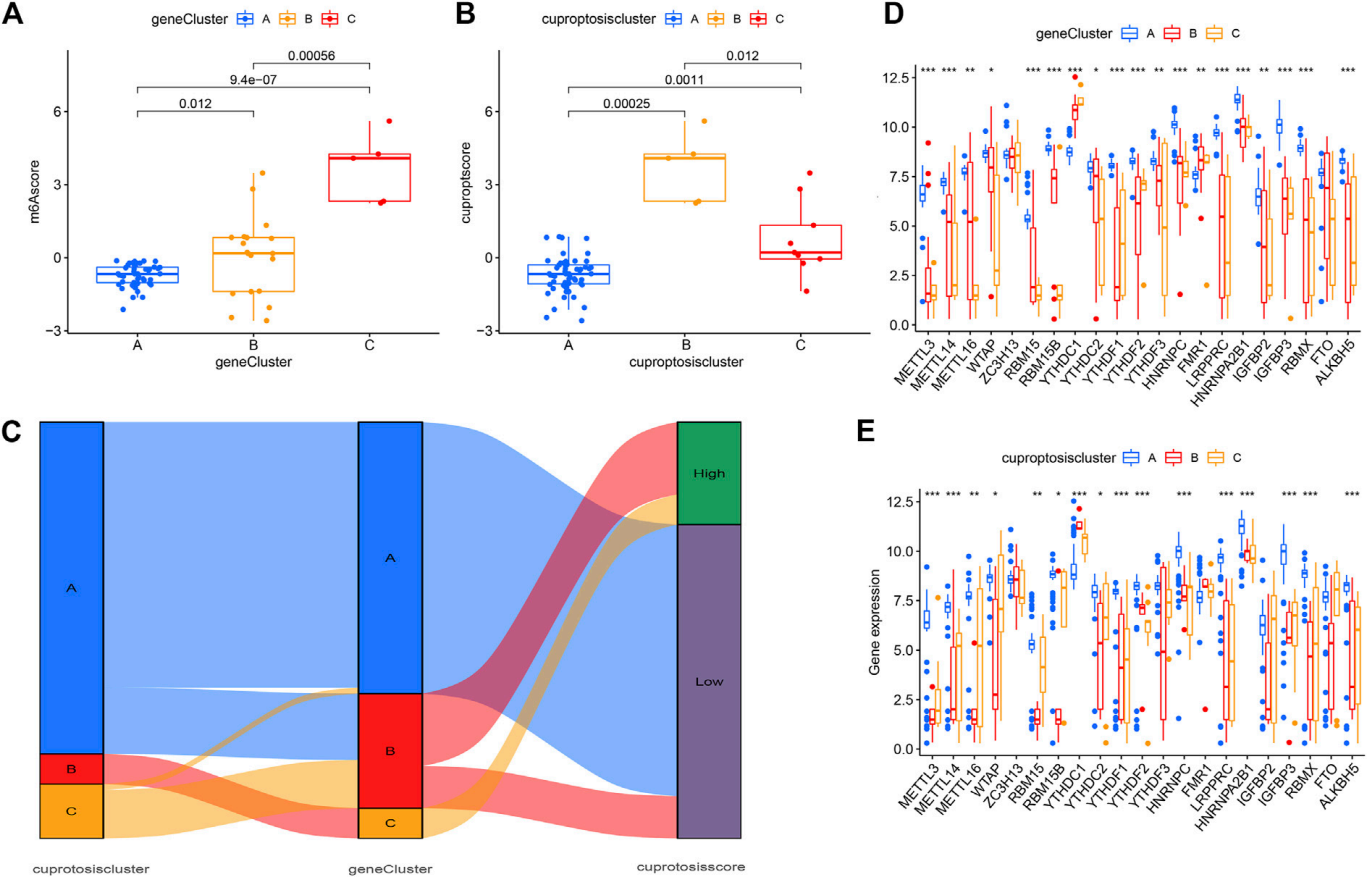
Roles of cuproptosis patterns in distinguishing psoriasis. **(A)** Differences in cuproptosis score between clusterA, clusterB and clusterC. **(B)** Differences in cuproptosis score between gene clusterA, gene clusterB and gene clusterC. **(C)** Sankey diagram showing the relationship between cuproptosis patterns. **(D–E)** Differential expression of 23 m6A regulators clusterA, clusterB and clusterC in gene pattern and cuproptosis pattern. *p < 0.05, **p < 0.01, and ***p < 0.001.

The results of consensus clustering analysis further validated the accuracy of our grouping. To quantify cuproptosis patterns, we used PCA to calculate cuproptosis scores for each sample. We then compared the scores between three different cuproptosis patterns or cuproptosis gene patterns. The results showed that in the gene cluster, the cuproptosis score of clusterC was higher than that of clusterA and clusterB (Figure 8A). However, in the cuproptosis pattern, the cuproptosis score of clusterB was the highest (Figure 8B). Finally, the relationship between cuproptosis patterns, cuproptosis gene patterns, and cuproptosis scores was visualized in a Sankey diagram (Figure 8C).

### The correlation analysis between cuproptosis-related genes and drug sensitivity

To further describe the relationship between cuproptosis patterns and the differential diagnosis of psoriasis, we conducted a correlation analysis between cuproptosis patterns and m6A. We tested 23 m6A genes and found that the expression levels of all genes in clusterA (except YTHDC1 and FMR1) were higher in the cuproptosis model than in clusterB and clusterC. This suggests that the cuproptosis pattern or gene pattern of clusterA and the m6A gene both affect the occurrence of psoriasis (Figures 8D, E).

### The correlation analysis between cuproptosis-related genes and drug sensitivity

**FIGURE 9:**
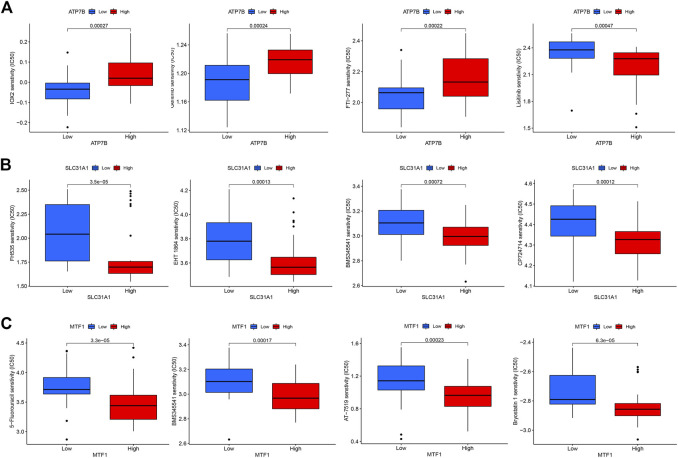
The drug sensitivity analysis of potential drugs for the treatment of psoriasis. **(A)** Sensitivity of ATP7B in different drugs. **(B)** Sensitivity of SLC31A1 in different drugs. **(C)** Sensitivity of MTF1 in different drugs.

To explore the correlation between key cuproptosis genes and drug sensitivity in psoriasis, we analyzed the sensitivity between ATP7B, SLC31A1, and MTF1 expression and drug sensitivity using the “pRRophetic” R package. The filter criterion was set to *p* < 0.001. The results showed that ATP7B, SLC31A1, and MTF1 were associated with susceptibility to 11, 16, and 30 drugs, respectively. These drugs are listed in Figure 9.

### Immunohistochemistry verification

**FIGURE 10:**
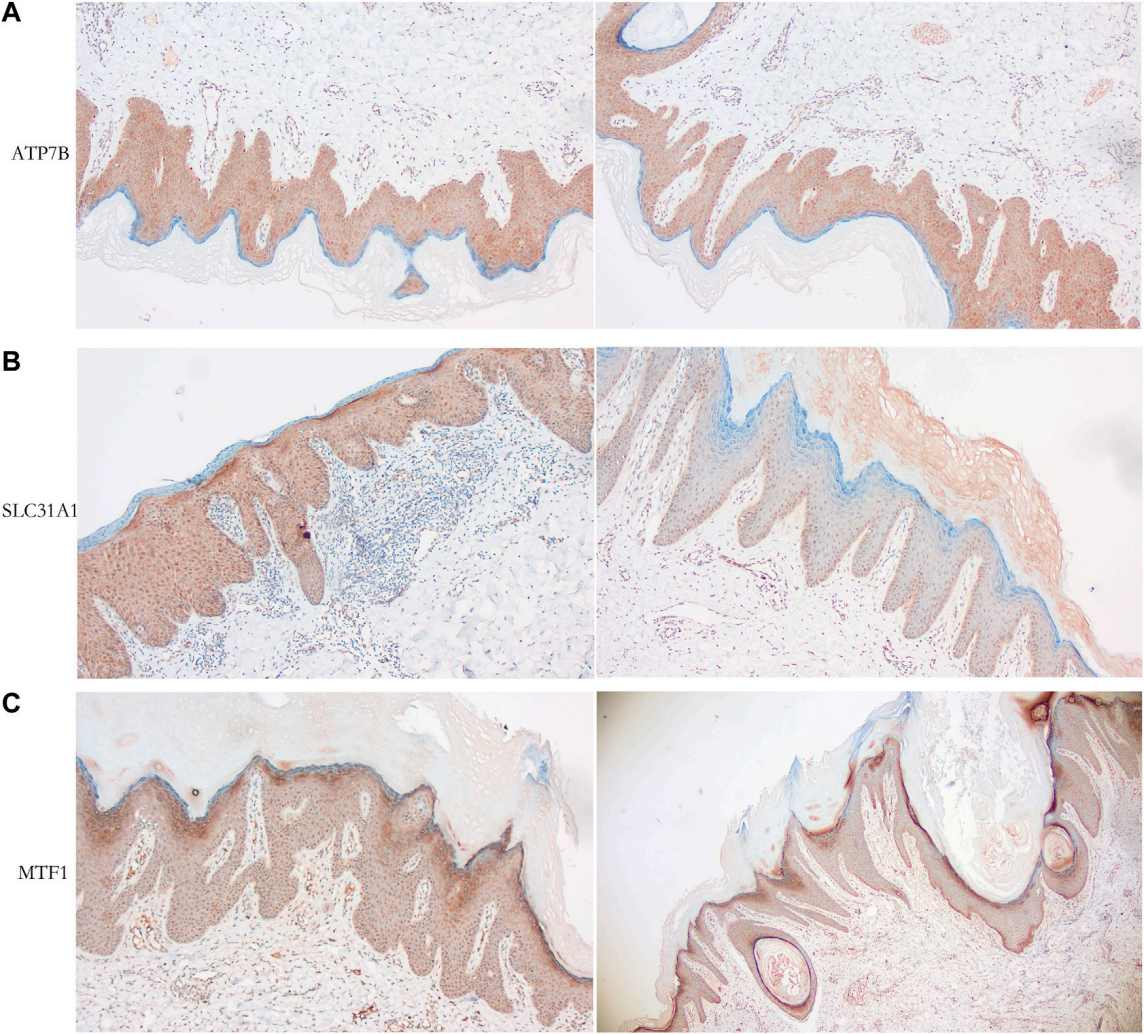
Immunohistochemical analysis of psoriasiss-related proteins. **(A)** Cytoplastic expression of ATP7B (tan particles of positive expressive object). **(B)** Cytoplastic expression of SLC31A1 (tan particles of positive expressive object). **(C)** Expression of MTF1 (tan particles of positive expressive object) stain the cytoplasm of epidermal insufficiency or hyperkeratosis cells.

We observed the expression of cuproptosis-related proteins, including ATP7B, SLC31A1, and MTF1, in psoriasis. The results showed strong cytoplasmic staining (Figure 10).

## Discussion

Psoriasis has always plagued many patients, affecting their quality of life. In some cases, it can even cause psychological problems, such as being afraid to talk to others and low self-esteem (Singh et al., 2017). Patients with psoriasis have different clinical presentations. The most common is chronic plaques or psoriasis vulgaris, characterized by well-demarcated, salmon-pink plaques with white skin. Other symptoms include guttate, erythrodermic, pustular, and silvery scales or gray patches on black skin (Griffiths et al., 2021). Genetic factors play an essential role in the pathogenesis of psoriasis. Psoriasis susceptibility 1 is a psoriasis susceptibility gene (Sagoo et al., 2004), and HLA-CW6 is another susceptibility allele of PSORS1 that is associated with the early onset and instability of psoriasis (Nair et al., 2006). Various driving factors can induce the occurrence of this disease, such as cadmium pollution (Liaw et al., 2017), ddrug-related effects (Balak and Hajdarbegovic, 2017) and streptococcal infection (Telfer et al., 1992).

Psoriasis is also a kind of common inflammatory skin disease. The content of copper in serum is a primary factor affecting the occurrence of psoriasis (Aggarwal et al., 2021). Elevated copper levels in serum may be responsible for the pathogenesis of psoriasis. Genetic variation in copper homeostasis leads to lethal disease, and excessive intracellular copper leads to cell death. Cell death induced by copper ionophores depends largely on the accumulation of intracellular copper. Studies suggest that FDX1 and protein fatty acylation are key regulators of copper ionophore-induced cell death (Tsvetkov et al., 2022). However, the mechanism of copper-induced cytotoxicity in psoriasis remains unclear.

In this study, we first identified the cuproptosis-related prognostic signature of 19 CRGs through differential gene expression analysis between non-psoriatic and psoriatic patients. Then, through establishing an RF model, we used the three candidate genes as cuproptosis regulators (MTF1, ATP7B, and SLC31A1) to predict the occurrence of psoriasis. The nomogram model using the candidate genes was also constructed. Based on the nomogram model constructed in this study, we found that DCA curve decision-making may benefit psoriasis patients.

The metal regulatory transcription factor 1 gene (MTF1) encodes a transcription factor that induces the expression of genes involved in heavy metal homeostasis in response to cadmium, zinc, copper, and silver. Studies have shown that MTF1 can bind to the metal response element to activate it and promote ATP7B gene expression to induce metal homeostasis (Giedroc et al., 2001; Stalke et al., 2020). Here, ATP7B is a member of the P-type cation transport ATPase family, which encodes an ATPase with multiple transmembrane domains and contains an ATPase consensus sequence, a hinge domain, a phosphorylation site, and at least two putative copper-binding sites. Mutations in ATP7B are associated with Wilson’s disease, a severe hepatic neurological disease. To date, hundreds of Wilson’s Disease-related mutations have been identified in ATP7B (Schushan et al., 2012). The protein encoded by the solute carrier family 31 member 1 gene (SLC31A1) is a high-affinity copper transporter found on the cell membrane, and the encoded protein acts as a homotrimer to affect dietary copper absorption. The expression of SLC31A1 is associated with a variety of cancers, including lung cancer (Wang et al., 2021), pancreatic cancer (Yu et al., 2019), and ovarian cancer (Wu et al., 2021). However, these three genes have currently not been reported for psoriasis. Our study could provide new directions for future experimental studies of cuproptosis regulators.

At present, most researchers are cognizant of the fact that m6A expression plays a regulatory role in the pathogenesis of psoriasis. Moreover, the overexpression of WTAP could be involved in the pathogenesis of psoriasis by regulating cell cycle progression. Thus, it is suggested that WTAP may be an underlying cause of psoriasis and a potential therapeutic target (Kong et al., 2020). IGFBP3 is a protease inhibitor in psoriasis, and changes in the IGF/IGFBP system may be involved in the pathogenesis of psoriasis (Xu et al., 1996).

In our study, three cuproptosis patterns based on gene patterns (ClusterA, ClusterB, and ClusterC) were identified from CRGs using consensus clustering. Most of the m6A genes tested were more significantly expressed in the cuproptosis and gene patterns in clusterA compared to clusterB and clusterC, suggesting that clusterA may be related to psoriasis. Further, this suggests that the cuproptosis gene and m6A both influence the progression of psoriasis. In addition, we conducted PCA analysis to calculate the cuproptosis score for each sample and found that clusterC had a higher cuproptosis score than clusterA and clusterB for the gene pattern, whereas clusterB had the highest cuproptosis score in the cuproptosis pattern.

Using drugs to treat psoriasis has been the main focus of our research. For this reason, we conducted drug sensitivity analysis on three essential cuproptosis genes. ATP7B is related to the sensitivity of 11 kinds of drugs, SLC31A1 is related to the sensitivity of 16 kinds of drugs, and MTF1 is related to the sensitivity of 30 kinds of drugs, so the relevant drugs can be selected to inhibit the cuproptosis gene. Unfortunately, we did not perform experimental validation of these drugs with essential cuproptosis genes due to limited conditions. We hope to continue conducting related research in the future.

In conclusion, this study systematically analyzed the landscape of molecular alterations and interactive genes of cuproptosis that may be involved in psoriasis. It successfully selected three CRGs that may play a crucial role in psoriasis outcomes, and we established a nomogram map model based on the cuproptosis genes to accurately predict psoriasis prevalence. The results also provide novel insights into the patterns of cuproptosis and may have the potential to aid therapeutic strategies related to cuproptosis for psoriasis prevention and treatment.

## Data Availability

The original contributions presented in the study are included in the article/Supplementary Material, further inquiries can be directed to the corresponding authors.

## Supplementary material

SUPPLEMENTARY FIGURE S1 The results of the permutation test.

SUPPLEMENTARY FIGURE S2 The correlation results between the genes.
