# Supplementary figures and images for: Significance of cuproptosis- related genes in the diagnosis and classification of psoriasis
Source: Front Mol Biosci. 2023 Apr 7;10:1115091. doi: 10.3389/fmolb.2023.1115091 (PMC10119406; doi:10.3389/fmolb.2023.1115091)

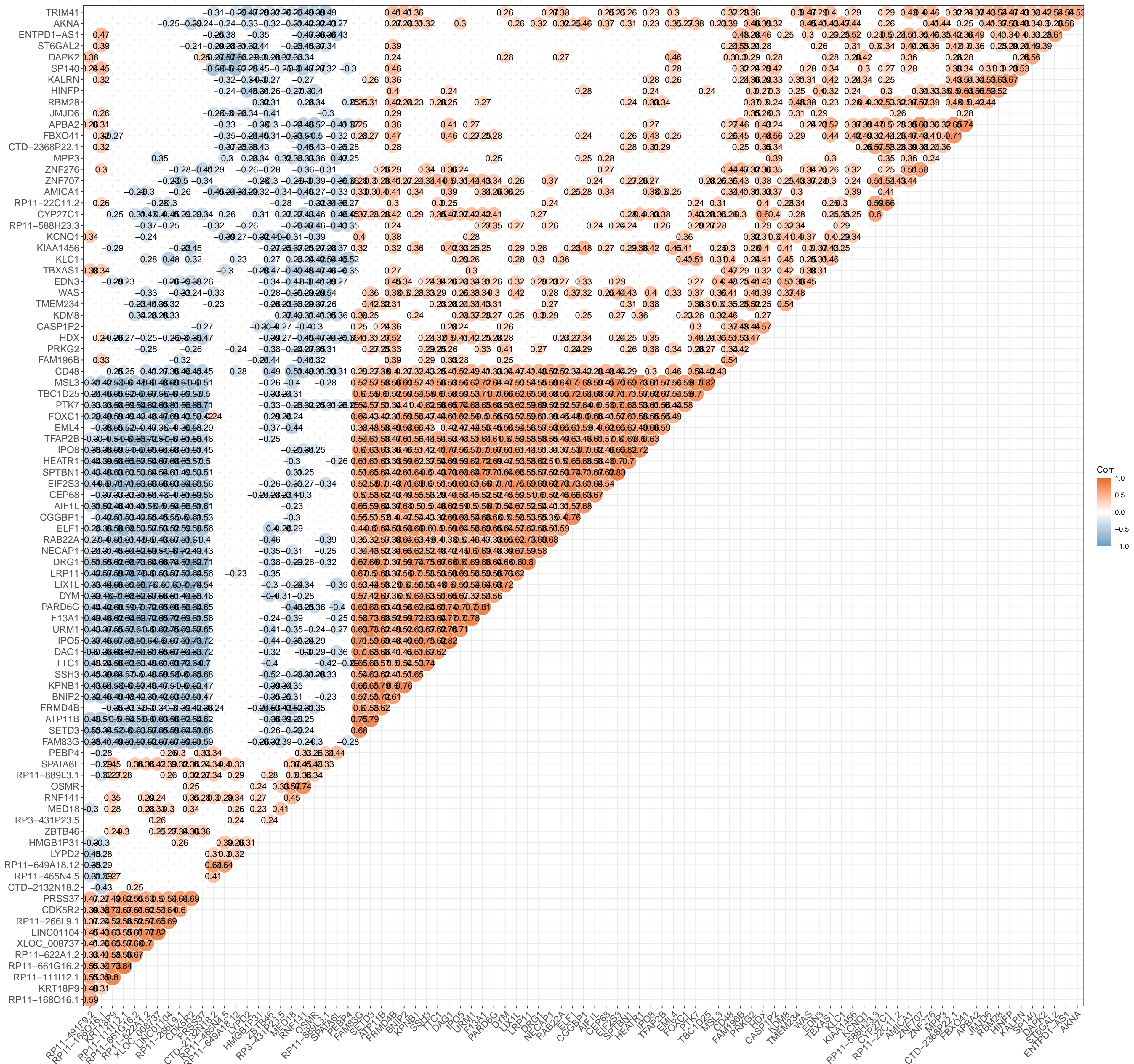

Supplement: Supplementary file 1 [file DataSheet2.PDF]
