# Supplementary material for: Significance of cuproptosis- related genes in the diagnosis and classification of psoriasis
Source: Front Mol Biosci. 2023 Apr 7;10:1115091. doi: 10.3389/fmolb.2023.1115091 (PMC10119406; doi:10.3389/fmolb.2023.1115091)
Supplement: Supplementary file 2 [file DataSheet1.PDF]

ATP78& SLC31A

list=[2.58991758e-01 4.30528426e-01 4.96974289e-01 6.47020597e-01
2.20755446e-02 1.77773164e-01 3.64613767e-01 3.66475225e-01
4.19674664e-01 3.31373057e-01 5.63711679e-01 1.07231282e-01
1.86035974e-01 1.21953360e-01 8.00046601e-02 3.76335585e-01
3.30311888e-01 1.33820407e-01 6.80370753e-01 6.26773956e-01
7.11158520e-01 7.35566979e-01 2.37917030e-01 6.21662518e-02
5.37492159e-01 3.30650382e-02 4.62841058e-01 1.90358851e-01
1.67642490e-01 6.78803012e-01 6.40949342e-02 1.02184326e-01
8.36451268e-01 3.05662191e-01 5.63876496e-01 4.00206065e-01
2.00735308e-01 2.76080320e-01 2.78071362e-01 4.24237287e-01
7.58332511e-01 3.94541591e-01 2.41008968e-01 2.06306415e-01
7.55931651e-02 4.04415616e-01 2.30483484e-01 3.70048469e-01
6.95638733e-01 2.96535234e-01 7.27007163e-01 5.44558902e-01
1.95427673e-01 1.35178016e-01 1.40189585e-01 5.55889230e-01
1.38587729e-01 2.46040575e-01 4.01640318e-01 7.85371173e-01
2.04264727e-01 1.39946267e-01 2.48594764e-01 4.28837345e-01
7.22931298e-03 1.03974554e-01 3.61174575e-01 3.34743537e-01
1.84296214e-01 3.61805259e-01 4.19301027e-02 3.93920853e-01
5.18979078e-01 3.17822040e-01 5.34272101e-01 2.47900622e-01
2.56429147e-01 3.65358657e-01 3.33423985e-02 3.87549463e-01
5.65996463e-01 5.32844744e-02 2.30678920e-01 1.17634113e-02
1.40575584e-01 3.68015782e-01 8.22234911e-02 8.82210894e-02
5.73876087e-01 9.19143065e-02 3.40159960e-01 2.00840714e-01
3.60164370e-02 6.48347115e-01 1.14572736e-01 2.37484175e-01
2.08953013e-01 4.00077520e-01 2.90229342e-01 4.79676732e-02
9.84767016e-01 4.60039038e-01 3.45796037e-01 2.79410631e-01
4.56941458e-01 2.51330316e-01 4.11892031e-01 1.72862866e-01
4.03722793e-01 3.89655280e-01 8.55789241e-01 2.81017949e-02
3.47350095e-01 3.33295943e-01 2.33351199e-01 1.43343290e-01
2.85070129e-01 3.36353230e-01 9.36308387e-02 2.11004345e-01
3.91445196e-01 2.22685511e-02 1.73130955e-01 1.93550276e-01
2.78694454e-01 7.46918520e-02 5.58983665e-02 2.46027912e-01
4.21099657e-01 4.67779066e-01 3.03813004e-01 4.39767396e-02
1.24022019e-01 3.11824289e-01 7.17537328e-01 1.54017117e-01
4.90439043e-01 1.24007280e-01 2.44859497e-01 2.72958851e-02
5.97015461e-01 3.75641800e-01 3.08604483e-01 6.40424470e-01
9.31815440e-03 2.11569455e-01 6.11060940e-01 7.63338694e-02

8.30447749e-01	1.22432416e-01	3.03362011e-02	4.64472861e-01
8.62599232e-02	5.82756305e-01	4.49339386e-01	4.81861394e-02
2.08406720e-02	4.95451972e-01	1.49815281e-01	9.57303845e-03
4.61276208e-02	5.07597142e-02	3.88665717e-01	7.99764294e-02
7.38774254e-01	6.93364982e-02	2.32595905e-01	7.23267756e-02
1.03692004e-01	3.78284840e-01	2.14406466e-01	3.12403398e-01
3.82486680e-01	3.01608959e-01	1.61025270e-01	4.97370551e-01
3.42618473e-01	7.38178401e-01	3.85757456e-02	1.17184170e-01
1.43771834e-01	8.48016107e-03	4.40197815e-01	6.86211854e-01
7.48854599e-02	2.80812169e-01	6.06990439e-01	3.04895581e-01
1.47263192e-02	1.37836142e-02	1.56855046e-01	3.37286345e-01
3.19778327e-01	3.95131422e-01	3.33167835e-01	1.93919996e-01
3.30513166e-01	1.53067538e-01	5.70738025e-01	5.06094959e-01
4.39184898e-01	3.74938749e-01	4.31306853e-01	1.07835038e-01
1.88549439e-01	2.75340763e-01	2.03936927e-01	2.64008718e-01
4.43176018e-01	5.74788097e-01	2.35605939e-01	3.60559777e-02
2.24708383e-01	9.86125665e-02	5.87325369e-03	5.79877878e-01
1.04709284e-01	4.51412489e-01	2.28621481e-01	2.58657987e-01
1.74300998e-01	5.84330018e-02	2.51824007e-01	4.98666085e-02
1.03481489e+00	1.52372192e-01	3.53032828e-01	3.52420830e-01
3.82389107e-01	1.62301388e-01	1.22385833e-04	1.37594541e-01
1.13887696e-02	3.88997087e-01	3.48532218e-01	2.81042908e-02
1.85020443e-01	4.59777475e-01	1.42215654e-01	6.03323182e-01
1.31084203e-01	1.95804756e-01	4.07925725e-01	1.72396787e-01
3.24719013e-02	2.03488730e-01	4.24197491e-01	7.76842134e-01
3.99534508e-01	7.61006898e-01	2.92871554e-01	5.13697959e-01
7.34888370e-02	2.48260509e-01	4.45971439e-01	2.54643942e-02
1.53216199e-01	1.23961024e-01	3.59439088e-01	1.97139177e-01
1.55041488e-01	2.29599920e-01	1.19997706e-01	3.58586927e-01
3.89190168e-01	1.56389895e-01	3.53647438e-01	1.45899289e-01
2.80141321e-03	3.47003819e-01	1.15315845e-01	4.65626752e-01
2.56649691e-01	2.51835641e-01	3.39313034e-01	3.56102935e-01
2.62305704e-01	1.14877734e-01	8.64527203e-01	2.05953942e-01
5.01046420e-02	1.39901861e-01	3.12043563e-01	8.78732477e-02
7.27298063e-01	6.21160316e-01	3.61883648e-01	9.07316027e-02
3.35158899e-01	1.20997578e-01	2.88992866e-01	1.42810165e-01
1.45595415e-01	2.09940101e-01	5.65878701e-01	1.00537704e-01
4.34162599e-02	5.24968411e-01	1.31480144e-02	6.01907622e-01
1.58293006e-02	2.93066778e-01	2.53041104e-01	5.17998582e-02
1.08070872e-01	5.72714735e-02	2.75474850e-01	9.18195923e-01
4.66931490e-01	2.22078609e-01	6.91551890e-01	1.00682413e-02
2.41996188e-01	4.83479472e-01	3.73178015e-01	3.76354446e-01
2.39679537e-01	4.89511515e-01	5.94508065e-01	9.32584454e-02

3.17170072e-01 4.33626983e-01 3.57283370e-02 1.34156444e-01
2.60189194e-02 1.97481266e-01 2.24302801e-01 4.79074581e-01
4.06407278e-01 6.36117380e-02 6.32995010e-01 5.63973346e-01
3.85204354e-01 7.15516902e-01 6.98641043e-01 4.88356900e-01
1.25874432e-01 2.28868928e-01 3.30720300e-01 3.53774880e-01
2.66816269e-01 1.83102036e-01 2.48351664e-01 2.34176865e-02
2.08312023e-02 7.55491050e-01 1.18368467e-01 4.25116005e-01
1.13585932e-01 1.30022248e-01 1.14066445e-01 3.91158333e-01
3.81380267e-01 3.11547594e-01 2.86182972e-01 2.48143375e-01
2.05402756e-02 1.72665547e-01 3.78756951e-01 1.27391289e-01
1.94174341e-01 6.82689212e-01 2.91103606e-02 1.40666305e-01
7.33076580e-02 1.02250675e+00 6.10115387e-01 7.45729494e-02
1.43007138e-01 6.15139996e-02 2.63409106e-01 2.05965163e-01
2.65077081e-01 2.94796697e-01 8.70512789e-02 3.38134907e-01
7.46915661e-02 2.06708734e-01 5.76077899e-01 1.66965703e-01
3.59555850e-01 1.41812615e-02 1.84894836e-01 5.91223459e-01
2.16882289e-01 3.23136176e-01 1.96449833e-01 1.18571468e-01
6.12380368e-02 1.04293760e-01 4.10546360e-01 3.36667996e-02
3.33074986e-01 1.02641765e-02 5.00869382e-01 8.95285558e-02
4.48381953e-01 4.61886669e-01 3.50143425e-01 3.63803436e-01
1.02972663e-01 1.42758449e-02 1.67511697e-01 3.55461553e-01
1.94362130e-01 7.76930770e-02 8.22879844e-02 6.72962350e-01
2.76991884e-01 1.67909827e-02 3.67880428e-01 7.29488051e-02
5.33341770e-02 3.17601944e-02 3.62290886e-01 7.52409113e-02
2.33109071e-01 3.15213491e-01 4.10925014e-01 1.08987489e-02
8.97505999e-02 5.56003339e-02 5.57687143e-01 3.89862530e-01
1.40714597e-01 3.29995231e-01 2.90484148e-01 7.48637065e-02
8.69886786e-04 7.83530061e-02 3.19576228e-01 1.11686161e-02
5.97663784e-01 3.43262758e-02 3.49973835e-01 7.52143089e-02
2.26642515e-01 4.88755942e-02 2.00937507e-01 2.16795960e-01
3.56405462e-01 1.92425142e-01 5.19849302e-01 1.56349329e-01
1.12732272e-01 5.03122557e-01 2.15004562e-01 4.04856744e-02
2.00809733e-01 6.28831548e-01 1.97176369e-01 1.80787911e-01
5.50697049e-02 5.17824577e-01 6.99785592e-01 8.20788489e-02
2.40848709e-01 3.29509119e-01 8.07060023e-02 3.20116977e-02
8.90116094e-02 4.27358164e-01 2.26942160e-01 4.69154052e-01
2.15432717e-01 1.86367051e-01 9.74645964e-03 8.15488720e-02
2.59460821e-03 1.86783639e-02 1.22156336e-01 1.26435708e-01
4.07661225e-01 1.32622707e-01 2.39801251e-02 3.67037272e-01
3.12032857e-01 3.82505459e-01 5.94978632e-02 9.31507746e-01
1.75172134e-01 2.38900669e-01 1.88329521e-01 2.20917128e-01
6.38776714e-01 1.61875586e-01 9.80221392e-02 9.75826489e-02
4.37280272e-01 4.06560835e-01 4.66278334e-01 7.71340232e-02

1.22681698e-01	2.50108767e-01	3.93537446e-01	7.54771909e-01
3.32808010e-01	6.55188473e-01	3.55953512e-01	1.43776467e-01
4.73752385e-01	2.86908734e-01	1.33249132e-02	4.32389540e-01
1.57003500e-01	3.46975293e-01	2.47296823e-01	6.26660573e-01
3.70847920e-02	1.53708880e-01	2.80668557e-01	3.79130398e-01
2.34876393e-01	3.80391756e-01	9.72128915e-02	3.46612150e-01
4.52100418e-01	5.76163501e-01	2.12739033e-01	1.81124922e-01
5.05324545e-01	2.41139046e-02	1.42866979e-01	5.22458883e-01
2.99437748e-01	5.45779242e-01	3.57567877e-01	4.66194889e-01
5.04813892e-01	3.14928946e-01	4.75593348e-01	3.74753884e-01
3.96870736e-01	1.85608613e-01	1.08786968e-01	7.34469480e-01
3.18560412e-01	2.23584006e-02	1.03481810e-01	1.41992293e-01
1.01988687e+00	5.19297483e-01	3.31942623e-01	1.53951941e-01
3.82232753e-01	1.52938449e-02	8.44818631e-03	2.52351113e-01
1.75437337e-02	1.41386079e-01	3.19575207e-01	1.97438917e-01
4.04232434e-01	1.88537702e-03	6.11712279e-01	2.82180541e-01
5.53463801e-02	3.38336726e-01	4.89732275e-01	5.45944746e-01
1.69653587e-01	4.11905171e-01	2.50727463e-01	1.36350879e-01
1.09467223e-02	2.56287247e-01	1.40993215e-01	4.72057614e-01
1.92576933e-01	6.37517879e-01	2.87658130e-01	1.01056138e-01
2.36283429e-01	3.96315859e-01	5.99498935e-02	1.87541763e-02
2.50847037e-02	4.76558037e-01	1.43092541e-01	2.47219765e-01
7.09337349e-02	1.83611796e-02	4.73987158e-01	1.35782663e-01
2.34201680e-01	8.83374228e-01	4.40872049e-01	7.13886858e-01
4.10659322e-01	1.45691039e-01	1.71073726e-01	4.90507252e-01
1.82201249e-01	5.59236435e-01	1.09736052e-01	6.65717957e-01
4.93899544e-01	1.10178856e-02	4.44082804e-02	3.24461283e-01
1.94864201e-02	6.96102946e-02	1.23606485e-01	1.34063917e-01
1.81461013e-01	4.27801724e-01	2.22596578e-01	1.94681873e-01
3.83416646e-01	2.65058842e-01	1.74401694e-01	2.65129340e-01
2.26098335e-01	2.01937481e-01	1.12804098e-01	3.74595451e-02
2.65146363e-01	2.11920512e-01	1.22760529e-01	1.47905131e-01
2.60020661e-02	2.28105799e-01	5.84914823e-01	1.24141422e-01
3.76522963e-01	3.66021085e-02	3.63920025e-02	4.44612040e-01
1.44656792e-01	2.20578938e-01	2.62015916e-01	2.61393086e-01
5.08539928e-01	5.27842602e-01	5.86282785e-02	4.54436573e-01
9.81915889e-02	3.84011827e-02	8.43187908e-02	6.43943877e-02
8.15836151e-02	4.33035376e-01	2.58571403e-01	2.28792743e-01
2.96279800e-01	4.06813937e-01	1.85047342e-01	6.49755725e-01
1.42780535e-02	1.99008004e-01	1.49615771e-01	2.37938784e-01
9.56163251e-02	2.65114698e-01	5.41594990e-01	2.67610162e-01
3.32244470e-01	4.06621080e-01	3.23794856e-02	4.74827790e-01
5.75898933e-01	8.82741430e-01	1.66004358e-01	7.71182011e-02

3.74239679e-01 2.76826722e-01 2.08633516e-01 2.26551938e-01
3.29550414e-01 3.12453863e-01 6.18226235e-02 4.81757513e-01
1.87611358e-02 3.92202491e-01 2.64668520e-01 1.27423285e-02
5.56529938e-01 3.76400230e-01 2.09993234e-01 3.01140213e-01
1.73487759e-01 1.41947735e-01 5.56039283e-01 4.39254399e-01
4.91834006e-02 4.88378359e-01 5.68529855e-01 2.75654751e-01
2.15775832e-02 3.80795539e-01 2.74770709e-01 3.70859261e-01
2.23194035e-01 7.48269555e-01 7.02166595e-01 3.82795420e-01
5.10527206e-02 4.74133923e-01 4.27415923e-01 3.18790151e-01
1.57904605e-01 2.84405527e-01 5.23521041e-01 4.23015578e-01
1.62871379e-01 5.10422083e-03 2.07714297e-01 2.27572849e-02
9.29608201e-02 1.77023390e-01 1.05393750e-01 8.61912496e-02
2.50167665e-02 1.69855913e-01 6.13694472e-01 3.16555673e-02
5.09434789e-02 4.98810692e-01 1.46062111e-01 2.10504356e-02
9.39092227e-02 5.21455571e-01 5.46687255e-01 4.72524817e-01
3.60332787e-01 1.86818783e-01 1.89552774e-01 1.31199189e-01
1.54992959e-01 1.45260824e-01 4.30441866e-01 7.57808489e-02
9.89928280e-02 2.11763115e-01 7.40291073e-02 6.84458997e-01
7.30163099e-02 3.35855492e-01 2.97521868e-01 2.59715587e-01
2.16250487e-01 5.12942681e-01 1.28030685e-02 5.34317861e-02
2.07724842e-01 6.80800275e-02 2.02528515e-01 2.72753849e-01
2.35187346e-02 7.69269732e-02 2.85087672e-01 7.53405673e-02
2.93568381e-01 7.35311108e-02 4.29843976e-01 1.14336645e-01
3.06765772e-01 3.94210752e-01 2.53418035e-02 4.83211272e-01
2.53586411e-01 1.73597714e-01 2.75519294e-02 2.11131890e-01
2.45248465e-01 3.51880022e-01 4.81319928e-01 1.74379874e-01
4.60519592e-02 1.07155463e-01 6.17691713e-01 3.55004701e-01
3.70421101e-02 6.95663465e-01 3.44065029e-01 2.73251936e-01
3.04445026e-01 2.64058840e-01 2.17486600e-01 2.60910083e-03
5.05215403e-01 3.10412555e-01 1.05437841e-01 6.97861591e-01
1.90855479e-01 4.08174142e-02 5.72285718e-02 4.40209169e-01
5.90831432e-02 3.11464126e-01 2.30360591e-01 5.89794878e-01
4.91850378e-01 3.95780874e-01 7.17400985e-02 3.31375627e-02
2.96000255e-01 2.33312018e-01 3.99765046e-01 7.61435356e-02
9.14474140e-01 2.42030941e-01 3.78451180e-01 1.80798776e-01
3.12280104e-01 2.35791945e-01 3.60890281e-01 3.21812360e-01
3.67457725e-01 3.88588890e-01 1.88795310e-01 3.97644555e-01
5.00660539e-01 4.09882677e-01 3.27699501e-01 3.65621997e-01
1.60701754e-01 6.05874842e-02 8.10796797e-01 1.35238110e-01
4.60115450e-01 2.43131489e-02 2.62547979e-01 4.51141563e-01
4.47099271e-01 5.50794089e-02 1.02190672e-01 3.52545067e-01
2.61117468e-01 1.16211555e-01 6.07450303e-01 3.11414274e-01
3.38045624e-01 1.47137113e-01 6.00741537e-01 1.38943289e-01

1.55421694e-01 4.36535756e-02 1.33857723e-01 4.86189500e-01
3.65999009e-01 4.84600454e-01 2.50147413e-01 2.12362049e-01
6.27805155e-01 1.23081412e-01 3.06057193e-01 7.99139118e-02
2.96169774e-01 1.94545419e-01 5.60878001e-02 6.71227863e-01
3.06176448e-01 1.29731850e-01 2.30170501e-01 3.98377815e-02
3.04202000e-01 5.20034088e-01 3.79190528e-01 1.09959686e-01
7.58276087e-01 4.58317930e-02 1.85917607e-01 8.33224371e-01
1.70724281e-01 3.05923924e-01 2.56345732e-02 1.48437749e-01
4.93232415e-01 1.28598478e-01 2.31647492e-01 4.53858943e-01
2.74445308e-01 3.07078827e-01 2.92771987e-01 7.29117945e-01
6.22600157e-01 2.13632045e-01 4.46312880e-01 1.30912622e-01
1.39553521e-01 6.32955654e-02 4.90593026e-01 6.79550821e-03
5.49128135e-02 2.17702095e-01 1.55114712e-01 5.63416643e-01
4.45962475e-01 6.01711215e-01 4.86322730e-01 1.04756908e-01
2.76947732e-01 1.06924854e-01 1.98173583e-03 2.35818706e-01
3.06235178e-01 6.09006364e-01 2.69061723e-01 1.19389982e-01
9.14756344e-02 2.75723618e-01 1.79071487e-01 7.11137668e-02
1.75692932e-01 3.94191883e-01 1.16507184e-01 1.91178445e-01
2.25115061e-01 7.19532023e-02 2.86903129e-01 3.91942921e-01
8.97423332e-02 2.04949583e-01 6.13439860e-01 1.17665938e-01
5.05493173e-01 5.09788183e-01 2.46378238e-01 2.79613693e-01
5.21741485e-02 5.16547194e-01 3.19168801e-01 1.05557137e-01
3.54084650e-01 3.93505387e-01 7.78502085e-02 6.24440799e-01
2.94204449e-01 9.60054296e-02 1.30868982e-01 5.86488272e-01
3.41355140e-01 5.79258637e-01 3.21050677e-01 1.42001531e-01
5.39422032e-01 8.36574463e-02 1.27801958e-02 1.40155489e-01
6.15428208e-02 7.92788127e-01 2.74693173e-01 1.28363253e-01
3.76804296e-02 1.06053803e-01 9.33248065e-01 4.43904667e-01
2.13687003e-01 1.46568307e-01 3.56864436e-01 3.80545098e-01
3.10973021e-01 3.45776179e-01 3.01815005e-01 1.90582288e-01
5.27860592e-01 9.94013701e-02 1.06115063e-02 4.43075901e-01
5.56892848e-01 2.29751373e-02 3.81783148e-01 5.69884088e-01
8.88376968e-02 1.76642473e-01 1.42885231e-01 7.83297503e-01
1.87214501e-01 4.90904971e-01 4.08783955e-01 2.16207298e-01
6.25970247e-01 3.14371414e-01 3.16249076e-01 4.00727786e-01
2.66726287e-01 2.70682382e-01 1.48610568e-01 4.76090668e-01
1.89880858e-01 2.48124995e-01 1.65565915e-01 6.64801364e-01
8.72660036e-03 1.07068373e-01 3.19677432e-02 3.32330791e-01
6.28682957e-01 6.29670737e-02 4.90245563e-01 4.76344463e-01
7.72447453e-01 4.91118224e-01 3.41857239e-01 2.01896429e-01
5.52884520e-02 2.98602373e-02 2.04513734e-01]

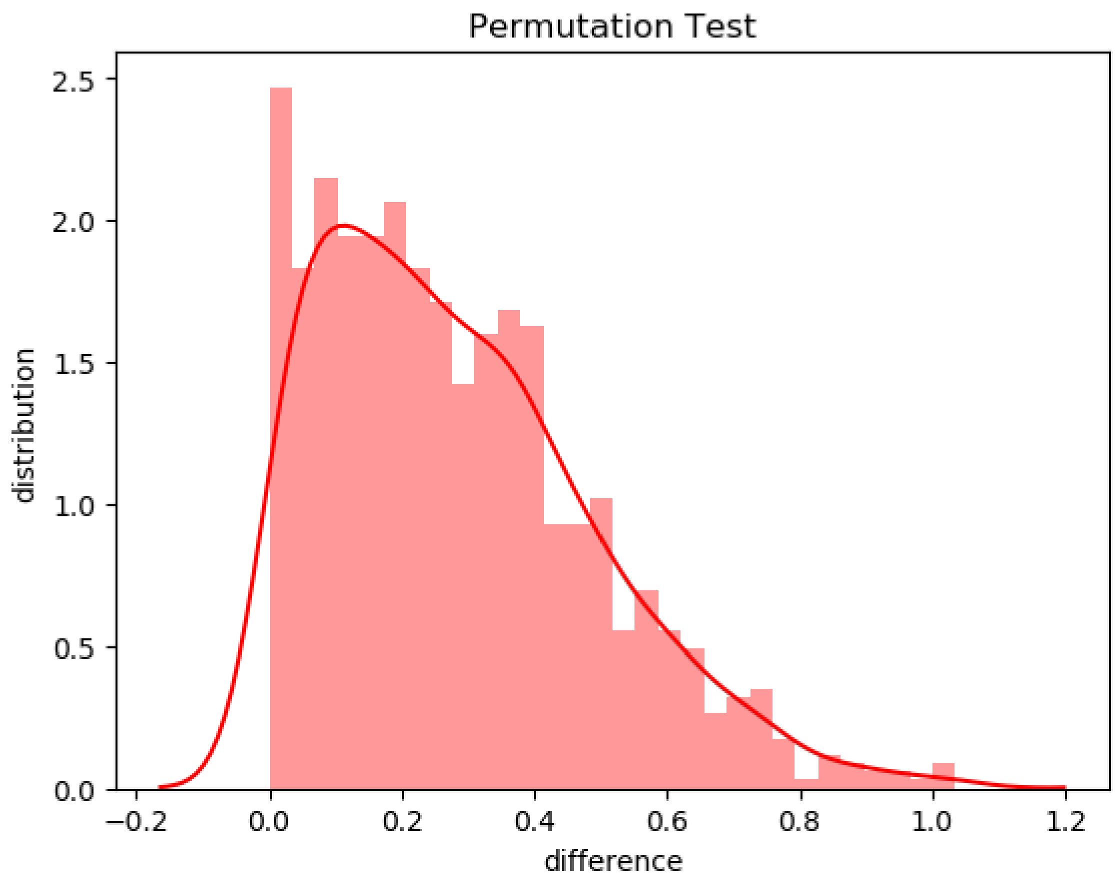

ATP78&MTF1

[1.59400016e-01 2.75587409e-01 2.22510965e-02 6.91183673e-01
7.00178689e-02 1.58902156e-01 1.36166059e-01 7.18470115e-02
6.20387626e-01 7.69854587e-02 2.80339263e-02 3.08515894e-01
2.71989093e-01 5.72117534e-01 3.89770238e-05 2.61327393e-01
6.92851506e-01 8.54768237e-02 5.09426194e-01 2.76414282e-01
1.71001703e-01 1.73619973e-01 1.26411782e-01 2.08687591e-01
4.55810788e-01 9.43502470e-02 3.12525879e-01 6.55832188e-01
1.22367772e-01 1.53002318e-01 5.81677365e-02 5.47611985e-01
1.72733881e-01 2.51836066e-01 4.37440992e-02 4.65765775e-01
3.68433508e-01 8.93708949e-02 5.35675164e-01 1.13716317e-01
1.87683701e-01 5.94192206e-01 1.53143140e-01 3.64829573e-01
7.07728830e-01 3.49111337e-01 9.93796073e-02 1.65269186e-01
1.19586689e-01 1.74096408e-02 1.54659597e-01 3.80293296e-01
2.54319533e-01 1.21325839e-01 1.53915955e-01 6.25429178e-01
3.01288532e-01 6.98114326e-01 7.91441532e-01 6.32595140e-01
3.68529321e-01 3.99709677e-01 1.70787678e-01 2.71107192e-01
2.23062342e-01 2.23473867e-01 9.95897180e-02 6.44936306e-02

6.55539656e-01 2.29821827e-01 3.77960817e-01 4.85458351e-01
2.07302196e-02 5.87440774e-03 5.98123632e-02 6.38705940e-01
9.10349042e-02 1.38673204e-01 4.20919368e-01 5.06315801e-02
3.83090123e-01 1.98469261e-01 2.80095555e-01 5.69701267e-01
2.68984451e-02 4.19920315e-01 2.78616872e-01 3.06177429e-01
8.40017935e-02 1.49015439e-02 1.53699617e-01 1.35523439e-01
4.49965659e-01 2.49299658e-02 4.15263137e-01 3.16769656e-02
2.23764390e-01 4.71803301e-02 1.56676722e-01 3.59126683e-01
3.14085761e-01 5.42156245e-01 2.80811703e-01 4.40887979e-01
2.79508160e-01 2.90639516e-01 6.03401104e-02 6.92021685e-01
3.19875036e-01 4.44022461e-01 5.62535532e-01 2.87737757e-01
9.19262958e-02 1.51698165e-01 8.10626794e-02 1.27723600e-01
2.09515458e-01 3.16042801e-01 1.23873400e-01 4.94940555e-01
2.51833995e-01 1.48760732e-01 1.26413401e-01 3.46863794e-02
6.23995615e-01 1.82429972e-01 1.97085203e-01 2.05541243e-01
5.59608751e-02 1.26232273e-02 2.26019651e-01 3.57573557e-01
3.04205973e-01 4.30309833e-01 3.27132246e-01 4.89795168e-02
5.81738806e-02 2.97899499e-01 1.60852350e-01 2.53584404e-01
7.04981310e-04 1.56781728e-01 1.93257744e-01 5.90652292e-02
5.61907253e-01 7.49973573e-02 2.18670354e-01 3.27194649e-01
4.02448549e-02 1.38275108e-01 6.14630877e-02 1.66624708e-01
1.88737505e-01 1.72787910e-01 3.24875200e-01 4.14424257e-01
1.21537631e-01 4.26942755e-01 2.84932278e-01 3.54568248e-01
4.49239901e-01 1.43596171e-01 2.38457623e-01 1.22172805e-01
2.27311469e-01 2.13836381e-01 2.36214750e-03 4.28562928e-01
3.02685696e-01 2.35093741e-01 2.72784922e-01 1.25899796e-01
2.52390825e-01 1.95826840e-01 9.64575301e-02 2.01145462e-01
3.76152966e-01 5.15635761e-01 1.92544433e-01 3.43134455e-01
4.93718236e-01 2.62971012e-01 1.63500678e-01 5.33829478e-01
2.51503832e-01 8.11411018e-02 3.60318127e-02 2.67277499e-01
2.07386454e-01 1.77492094e-01 3.34880394e-02 6.40103973e-01
1.48080057e-01 1.77030472e-01 9.02508332e-02 2.61067466e-01
2.21126608e-02 3.45406399e-01 4.02632346e-02 4.50752340e-01
3.58870679e-01 5.74703218e-02 2.86062937e-01 4.95628115e-02
3.63800187e-01 1.76112592e-01 1.36668850e-01 4.09960327e-02
2.11273104e-01 2.27059894e-01 2.35554623e-01 5.75539473e-01
1.08136051e-01 2.18645640e-01 7.24090194e-02 4.21680685e-01
1.95629838e-01 6.47360406e-02 2.28744107e-01 5.75700292e-02
3.68633559e-01 3.21874446e-02 1.38275845e-01 2.11246113e-01
1.44139709e-01 2.26460094e-01 4.45626870e-01 4.73652234e-01
5.52606659e-01 2.98625510e-01 5.39608138e-01 2.20141196e-02
1.22965695e-01 1.03524154e-02 5.41392527e-01 2.27694681e-01
2.40226778e-01 4.87306807e-01 2.72442059e-01 1.38870052e-01

5.64723712e-01 1.67110845e-01 2.80117340e-01 4.96523006e-02
1.82831358e-01 4.93031966e-01 1.25360431e-01 9.77422108e-02
3.06385403e-01 1.21783793e-01 8.46184468e-02 1.42916931e-01
1.72357975e-01 2.00351594e-01 7.81429451e-01 1.09702494e-01
2.26361377e-01 1.25647555e-01 4.34127689e-01 2.91130004e-01
5.02308496e-02 1.54121281e-01 3.72190639e-01 9.40507758e-02
3.54926573e-01 1.95934843e-01 1.16041286e-01 2.56001457e-01
3.69315522e-01 7.42743365e-02 3.12745508e-01 3.71780708e-02
1.20555840e-01 2.31093055e-01 7.76663756e-02 3.84910668e-02
9.73207556e-02 2.70655393e-03 6.58658054e-02 1.16088558e-01
2.71305405e-01 3.54073872e-01 4.91338567e-01 3.42778327e-01
3.55995067e-01 2.94849801e-02 1.93298025e-01 2.42473833e-01
4.92554857e-01 5.28236918e-02 1.97779225e-01 2.52918097e-01
2.95524368e-02 6.07205960e-01 3.71377274e-03 2.23139823e-02
2.65403163e-02 3.34259190e-01 6.00074536e-01 1.73585422e-01
9.91199139e-02 3.71827043e-01 1.81906531e-01 2.19404245e-01
5.70216516e-01 3.10383840e-01 5.06928236e-01 1.63618770e-01
2.50744821e-01 2.35084660e-01 1.03394169e-01 5.69280725e-02
5.38649670e-02 2.03725485e-01 3.47514335e-01 2.91078831e-01
3.47378877e-02 3.08752723e-01 3.47903208e-02 6.69218754e-01
6.12431954e-02 5.04928937e-01 3.82613269e-01 3.60799365e-01
4.98997266e-01 3.74921064e-01 1.79399836e-01 6.10993564e-01
1.00376723e-01 3.26573382e-02 2.80008905e-01 3.37769488e-01
3.58740656e-01 2.63446533e-01 2.23438818e-01 1.30917514e-01
2.59934091e-01 2.69298685e-01 1.94434498e-01 5.52650792e-02
1.87043425e-01 5.77667846e-02 2.00358446e-01 1.62114860e-01
2.00411130e-01 4.97844540e-01 2.75706327e-01 2.63817498e-01
6.78842620e-02 1.81110869e-01 2.69612000e-01 2.19554532e-01
8.04866123e-02 1.31207808e-01 1.79074708e-02 1.06522366e-01
1.75148063e-02 4.11724776e-01 1.48759348e-01 2.03743455e-01
2.65354743e-01 1.67902625e-02 1.14890888e-01 1.07172158e-01
8.22250196e-02 4.28592985e-02 4.61321166e-01 2.27469311e-01
2.69052687e-01 3.84773910e-01 3.00443953e-01 3.91077521e-01
5.91387748e-01 1.40412296e-01 8.13517140e-01 2.62896105e-01
1.64789947e-01 4.12528767e-01 1.12418370e-01 2.54558666e-01
2.31083944e-01 3.17675296e-02 1.71219204e-01 2.02651240e-01
9.02848213e-02 7.47051258e-02 3.71420682e-01 1.28879124e-01
1.76251802e-01 2.05947561e-01 5.26837440e-03 5.26378691e-01
1.17466782e-01 6.04692847e-01 2.32183612e-01 4.31308152e-01
1.41238980e-01 3.68595362e-01 2.32431618e-01 2.65308086e-01
7.56400911e-02 5.41365075e-01 5.11902552e-01 3.48320835e-02
3.44282542e-01 3.61872618e-01 3.72606123e-02 1.25573045e-01
8.50154051e-02 2.02834033e-01 2.41790858e-02 1.47813012e-01

9.38609589e-01 1.34146985e-01 2.57388264e-01 1.49182765e-01
2.98611006e-01 4.05893840e-01 1.66624594e-01 5.25835390e-01
1.95070903e-01 6.03273212e-01 7.14358782e-01 7.23362642e-01
1.92234676e-01 1.34490668e-01 2.15189332e-01 1.24285443e-01
5.72245945e-01 3.63674738e-01 2.93006154e-01 1.79796565e-01
1.68114386e-01 8.44159670e-02 7.07140242e-02 4.01070123e-02
1.55739067e-01 3.32483802e-01 1.69900074e-01 2.74762554e-02
5.73667181e-01 2.13072193e-01 7.66240975e-02 8.91059837e-02
1.83913644e-01 4.34242677e-01 6.11486675e-01 1.06190695e-01
4.69536711e-01 8.94810893e-03 4.39003728e-01 6.49475238e-01
1.00503301e-01 1.40185824e-01 2.36036987e-01 2.58273592e-02
2.99890413e-02 2.26846239e-01 5.62697192e-01 4.24477231e-01
1.86392777e-01 2.02234015e-02 2.23070009e-01 4.10353195e-01
1.54747607e-01 6.92995435e-01 1.95434677e-01 1.27443741e-01
1.90319180e-02 3.35197385e-02 4.52337241e-01 1.58387388e-01
4.56823056e-01 1.22831753e-01 7.29835463e-02 7.22165132e-02
7.71084094e-02 1.92961899e-01 1.74907385e-01 7.66438175e-01
1.12963695e-01 9.35589701e-02 1.20396587e-01 3.41618719e-01
1.75946057e-01 1.71430823e-01 1.59958245e-01 5.24107556e-01
1.31191546e-02 3.57173775e-01 2.43905389e-01 4.73522483e-01
7.03056774e-03 4.47712904e-02 4.06891561e-01 2.02674894e-01
4.69567560e-01 1.05154975e-01 4.19710068e-02 1.82365902e-01
3.13080553e-01 6.29618102e-01 4.03838425e-02 8.97918263e-02
2.61230277e-01 1.18094481e-01 2.45080832e-02 4.37538837e-01
3.41514716e-01 4.91815395e-01 1.60338509e-01 4.63833084e-01
6.30029875e-02 2.82889037e-01 3.97817262e-01 1.09447453e-01
1.77527415e-01 1.29026598e-01 6.82658946e-02 2.95265104e-02
3.83274034e-01 5.75232048e-01 3.59129836e-01 5.51111646e-02
2.69795965e-01 4.50175192e-02 4.89033233e-01 7.79050118e-02
1.35403012e-03 1.51772329e-01 9.04978818e-02 7.27973727e-02
2.52305682e-01 4.07545284e-01 3.84194806e-02 2.90320018e-01
2.84780332e-01 1.82303497e-01 9.54296675e-02 2.34516528e-01
5.83451020e-01 4.21132468e-02 5.47854842e-02 6.94540151e-02
8.95771870e-01 1.07573145e-01 8.89848313e-02 2.03887687e-01
3.19811515e-02 3.29138255e-01 3.55441606e-01 3.92936016e-01
7.15142730e-02 2.18537415e-01 8.42117042e-02 4.66796108e-02
3.84810274e-01 5.97696216e-01 1.16621539e-02 8.62309406e-02
3.16041173e-01 3.90703387e-01 7.55604837e-02 3.81113673e-01
5.41529337e-02 5.17523030e-02 1.69498799e-01 1.66261747e-01
3.04918588e-01 1.18015335e-01 3.80452703e-01 1.05690527e-01
1.63860022e-01 2.20674955e-01 4.75362612e-01 7.19103392e-02
1.95942354e-01 2.14203005e-01 8.03058280e-01 6.01348859e-01
1.36699887e-01 2.01810036e-01 2.77436191e-01 2.52512727e-01

2.06927410e-01 2.03518870e-02 1.26057859e-01 4.11866255e-01
4.71850552e-01 3.08075138e-01 1.02722798e-01 3.16871970e-02
8.35779949e-02 1.32550782e-01 3.13256029e-01 1.72748487e-01
7.28443254e-02 6.38273289e-02 8.69867289e-02 1.26928966e-01
7.69638940e-03 3.34358569e-01 2.24380930e-01 5.50965681e-01
1.67348942e-02 3.57427199e-01 7.70145109e-01 4.91402221e-01
1.89613691e-01 7.17740725e-02 2.17847167e-01 7.00480810e-01
1.77276961e-01 3.48496992e-02 3.80934726e-03 8.10726548e-01
5.29993191e-01 2.39583952e-01 8.27274127e-02 3.46473733e-01
4.17540213e-01 3.80362912e-01 2.13294751e-01 4.22434687e-02
6.70395673e-02 4.75130575e-01 2.31423231e-01 5.75726042e-02
2.45114195e-01 4.99855347e-01 4.55456065e-01 6.43055620e-01
2.57130732e-01 5.98275287e-02 3.38540168e-02 2.58475706e-01
2.46128733e-01 8.66016070e-02 4.04333716e-01 6.33222080e-02
1.07535764e-01 2.43194165e-01 4.05455821e-01 3.20763621e-01
1.58310557e-01 8.48689945e-01 1.52980833e-01 3.01669922e-01
9.35792161e-02 3.62991163e-01 2.59098346e-01 2.63020368e-01
4.35762707e-01 1.36430286e-01 1.14843564e-01 1.12899722e-01
1.20465018e-02 7.12334192e-01 2.40377145e-01 4.58688060e-03
3.05100361e-02 4.16394065e-01 1.37688354e-02 1.80552372e-01
9.43229318e-02 3.15841046e-01 5.04414487e-01 6.57720905e-01
3.10818902e-01 4.42061025e-02 3.98183250e-01 1.00089644e-02
2.08539658e-01 2.19421925e-02 5.45385452e-01 3.96562023e-01
8.41298811e-02 1.15094920e-01 5.68744213e-01 6.35422697e-01
6.59496788e-01 4.26674117e-01 1.42763467e-01 4.92988740e-01
4.18853284e-01 3.15640187e-01 1.13695827e-01 2.59716144e-01
4.39779321e-03 3.38363922e-01 7.60660745e-01 7.19150742e-01
1.71666971e-01 5.80483814e-01 4.60462077e-02 1.52005913e-01
3.62452351e-01 1.08460088e-01 1.00717935e-02 5.08328478e-01
4.76278389e-02 3.13948596e-01 1.87522429e-01 5.81844241e-01
5.82949183e-01 1.15356921e-01 9.91557146e-02 2.21289885e-01
2.08459508e-01 2.84749553e-01 2.37603976e-01 3.28431746e-01
4.59849672e-01 5.97947006e-02 2.15103213e-02 1.36816057e-01
4.53755939e-01 3.23073819e-01 1.57677613e-01 8.70996939e-02
9.86032839e-02 1.44421895e-01 5.47018729e-01 2.18363512e-01
3.07122244e-01 7.08635935e-02 3.03624738e-01 2.42838958e-01
2.72821748e-01 3.12584447e-01 6.83100015e-02 7.17403553e-01
3.11402986e-01 4.13736256e-02 2.32079269e-01 4.92348090e-01
2.59720363e-01 4.08037920e-02 2.33274380e-01 1.53957768e-01
1.11911777e-01 3.77056162e-01 1.05472902e-01 5.85540776e-01
2.04937578e-01 4.72934606e-01 2.69587322e-01 1.90091777e-01
7.00671737e-02 3.59666337e-01 1.03351263e-01 3.98767788e-01
1.74514906e-01 7.92689772e-01 1.36375618e-02 1.54281759e-01

2.01581448e-01 4.06055780e-01 8.48111013e-02 6.69459699e-01
2.69725725e-01 1.35885679e-01 6.97183478e-01 1.61943697e-01
3.44072130e-01 1.05367284e-01 4.56749321e-01 2.86657023e-01
9.09400365e-02 1.03077383e-01 4.41072595e-01 1.71121312e-01
3.40204137e-01 3.65968349e-01 5.14242238e-01 4.17065586e-01
3.00706507e-01 4.58040128e-01 1.03573549e-01 5.58212004e-01
1.49016865e-01 1.46500923e-02 6.84634367e-01 1.67192236e-01
1.30177938e-01 3.18441037e-01 2.57500745e-01 2.65507292e-02
1.44518517e-01 7.05352327e-02 4.23310030e-01 6.59134744e-02
8.10914096e-02 2.12046456e-01 3.44951604e-01 3.20625622e-01
1.75711283e-01 7.26214937e-02 5.33967856e-01 1.35526712e-01
2.22059947e-01 2.48690714e-01 4.28414893e-01 8.48863020e-02
3.69215363e-02 2.91920808e-02 6.77395758e-01 1.22566308e-01
1.25514301e-01 1.93865198e-01 3.83511693e-01 2.48531887e-01
2.10713105e-01 2.89158232e-01 1.31152763e-01 3.07695782e-01
3.17386527e-02 8.31765339e-02 1.29749784e-01 4.76574891e-01
2.99724483e-01 1.62970930e-01 8.25981989e-02 1.97941975e-01
2.06074819e-01 2.80988696e-02 4.31450278e-01 4.53518214e-01
1.78784799e-01 2.48112328e-01 9.06964075e-01 3.59667799e-01
2.34753589e-02 2.16707387e-01 3.29523990e-01 4.47406761e-02
2.95073964e-03 6.10725867e-01 6.62735235e-02 3.60507594e-01
1.72347117e-01 5.74128345e-01 1.45461491e-01 1.47942762e-01
3.86273231e-01 2.23689901e-01 1.37637830e-01 4.23021201e-01
3.27778155e-01 7.50332937e-02 2.59742390e-01 9.47610794e-02
4.85341591e-01 2.67517895e-01 2.92698919e-01 2.18475287e-02
2.40176073e-01 9.50040235e-02 4.40821625e-02 2.93195422e-01
2.36395546e-01 1.04492507e-01 2.17588668e-02 3.94581018e-01
3.84424457e-01 8.64984435e-02 3.46814400e-01 3.88906371e-01
8.63729935e-02 2.66814744e-01 7.05442625e-02 5.32677639e-02
1.71548530e-01 6.95908096e-01 3.03614807e-01 1.28856391e-01
1.32045274e-01 1.03843574e-01 2.73813599e-01 6.75229701e-01
7.55993775e-02 1.67353908e-01 1.57253283e-01 1.82690375e-01
1.01433678e-01 1.58855592e-01 5.13099516e-01 1.81054611e-02
1.36249681e-01 4.31049266e-01 5.97904008e-02 5.91992339e-02
3.07095842e-01 1.08061838e-01 1.32352254e-02 3.53855589e-01
1.44874332e-01 3.36255780e-01 1.59872839e-02 3.92690350e-01
2.83585918e-01 7.10454796e-02 1.28876579e-01 2.40183247e-01
1.32010384e-01 1.05942781e-01 2.29935757e-01 2.34416368e-01
2.56619768e-01 2.55974913e-01 4.54368423e-01 1.78271377e-01
3.34737007e-01 1.46309456e-01 3.21852227e-01 2.73071473e-01
3.16188481e-01 2.69682663e-01 3.25832473e-02 1.16762089e-01
6.57287835e-02 1.84950562e-01 3.64097788e-01 6.84121165e-02
1.71842256e-01 4.83601936e-01 2.05351983e-01 1.41161587e-01

4.58803272e-01 1.99910749e-02 1.12200249e-01 3.83383824e-01
5.94614872e-01 4.83060760e-01 2.85355995e-01 1.80555112e-01
2.17993511e-01 1.56319163e-01 2.28559575e-01 2.80024586e-01
9.93938246e-01 1.34658055e-01 2.47695233e-01 3.22349176e-01
2.21249864e-01 1.22045799e-01 3.26718330e-01 2.03074225e-01
4.54273086e-01 6.12016470e-01 2.90618397e-01 8.26045546e-02
4.59678777e-02 6.68853254e-02 3.95261372e-01 3.21019817e-01
6.16459641e-01 4.77612672e-01 9.05237821e-03 1.77039440e-03
2.23363747e-01 8.59109387e-02 3.50607079e-01 5.54538301e-02
1.56375351e-01 1.03823083e-01 3.15935056e-01 1.54743658e-02
3.03602562e-01 9.69112856e-02 1.41200919e-01 6.44475488e-03
4.60710564e-01 9.42984249e-01 4.39562880e-02 3.19230222e-01
3.40424497e-01 3.01730782e-02 5.04164898e-01 8.71246585e-02
5.51139437e-01 1.49452659e-01 2.22368007e-01 5.10891039e-02
1.17956314e-01 2.13030778e-01 1.77440427e-01 7.63930294e-02
3.29586251e-02 1.26501871e-01 5.23516001e-02 1.03324801e-01
3.74903554e-02 2.69132125e-01 3.66397209e-01 2.66446876e-01
3.45818443e-01 2.21929537e-01 5.23931629e-01]

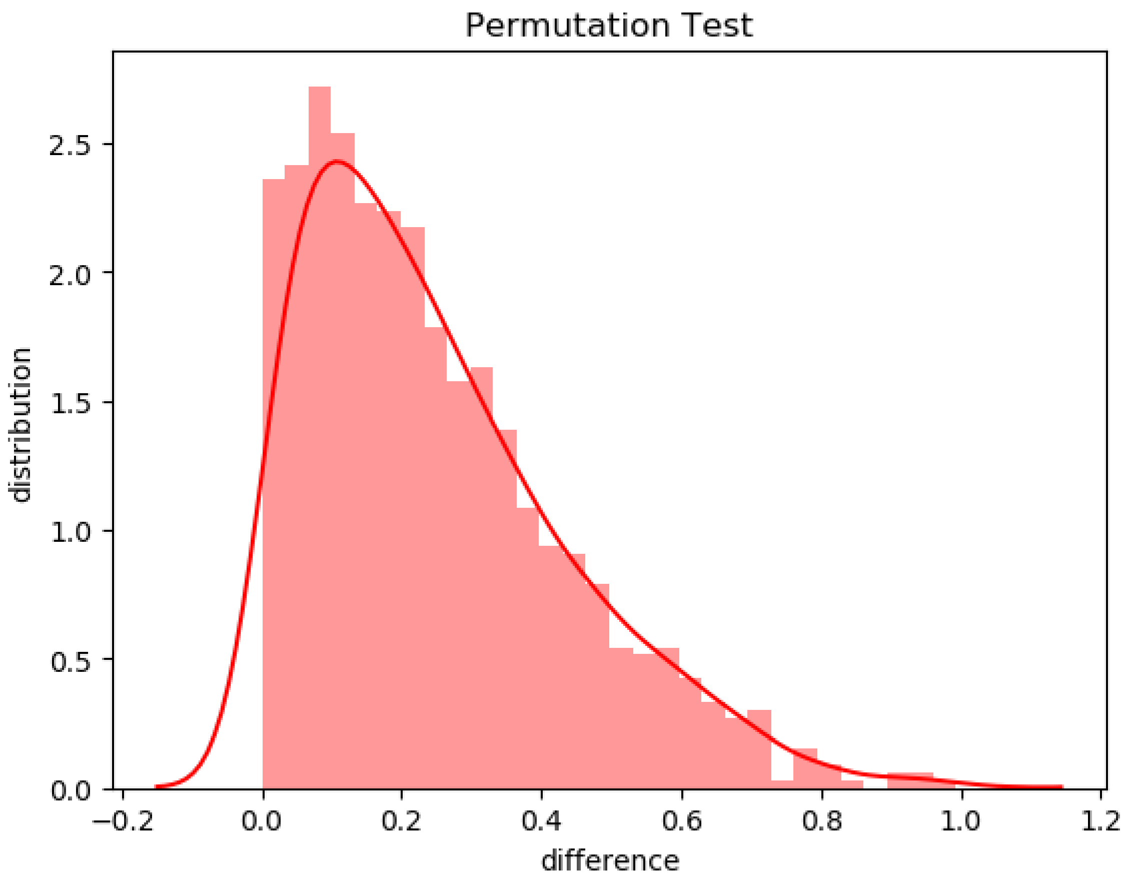

MTF1&SLC31A

[8.55496133e-02 3.05238527e-01 2.10304424e-01 2.92291543e-01
8.24819147e-01 8.63479224e-02 1.98952175e-01 2.49134673e-01
8.27267019e-02 3.69546209e-01 4.30459358e-01 1.42388565e-01
1.72457545e-02 2.58107919e-01 3.83094982e-01 3.07491009e-01
4.23307318e-01 3.82490163e-01 1.46267307e-01 2.86141309e-01
1.12797354e-01 7.06497398e-01 1.14905016e-01 2.07905540e-01
2.80233408e-01 1.52482943e-01 4.48262143e-01 8.83195476e-04
1.12819291e-01 8.29690183e-02 1.26839281e-01 3.24618106e-01
2.64500312e-01 1.79984006e-01 1.55608655e-01 1.46079321e-01
3.16145871e-01 4.57844550e-02 2.26388027e-01 2.42645720e-01
3.25073441e-01 4.46303019e-02 2.13219902e-01 1.18953716e-01
9.78787840e-02 1.25134993e-01 1.24445985e-01 1.50583635e-01
2.53843151e-01 1.04862239e-01 2.04066767e-01 1.97472177e-01
7.51158702e-02 7.98794245e-02 9.62144736e-02 3.29046656e-01
6.14328400e-02 3.93255249e-01 1.06704662e-02 1.89232686e-02
3.06780095e-01 1.17693628e-01 5.87642310e-03 1.12676483e-01
4.04442608e-01 3.74240554e-01 1.96519634e-01 1.86511599e-01
3.79912203e-01 3.62784860e-01 4.63030034e-01 1.88094305e-01
4.38573468e-01 3.70191551e-01 3.44261039e-01 6.59906460e-02
1.40371159e-01 2.34014776e-01 1.09876084e-01 4.59451021e-02
2.74488830e-01 6.06491443e-02 4.70178414e-02 1.89069069e-01
3.75585493e-02 5.35127957e-02 1.16500867e-01 3.38291025e-01
3.94698238e-02 4.05685744e-01 5.62791221e-01 3.71590102e-02
6.28235352e-01 5.94767836e-02 1.71013774e-01 2.45147565e-01
4.14815971e-02 2.42239773e-01 2.80446579e-01 2.11606600e-02
2.79185953e-01 4.83783413e-01 4.40196948e-01 7.46550234e-01
2.58539860e-01 4.15157301e-01 3.37527941e-01 4.26350310e-01
1.80826876e-01 3.02479261e-01 1.20162818e-01 1.23358848e-02
4.55407810e-01 4.22754606e-01 2.96766602e-01 1.18579500e-03
3.08408890e-01 1.39140068e-01 1.58767659e-01 3.00875866e-01
1.47675684e-01 1.55969660e-01 2.38382369e-01 1.15141450e-01
5.61438731e-01 2.41628652e-01 6.95671850e-02 2.59312892e-01
2.53175950e-01 2.56312094e-01 2.07181894e-01 6.54845159e-01
2.32949380e-01 2.96978810e-04 3.08589278e-01 3.49197828e-01
3.80088240e-01 3.36893135e-01 1.11934279e-01 4.40070221e-01
5.21322679e-01 1.55268133e-01 1.34529744e-01 8.62780788e-02
2.87217892e-01 8.19189530e-01 1.55858744e-01 2.44411169e-01
6.56789136e-02 1.92532031e-01 2.24073199e-01 2.49779025e-01
2.18734421e-02 1.33015002e-01 1.33329509e-01 1.24316275e-01
1.92099123e-01 4.10588147e-01 2.73081096e-01 5.43931174e-02

3.75526095e-01 4.54077986e-01 3.79243809e-01 2.11363850e-01
2.06028167e-01 4.85295934e-01 1.46824467e-01 1.00373029e-01
1.54564600e-01 2.93046275e-01 8.81457229e-02 3.54502790e-02
1.45715056e-01 4.82237405e-01 2.50226099e-01 1.45735019e-01
3.74923867e-02 3.61810855e-02 7.00150645e-02 6.37059612e-02
3.72115965e-01 2.42557568e-01 5.22198342e-01 1.03307183e-01
4.54195967e-01 3.69596512e-02 1.75762230e-01 3.18350133e-02
8.04816903e-01 1.42009181e-02 2.35165579e-02 7.68413621e-02
9.08929298e-02 4.01308913e-01 2.99026050e-01 2.74217050e-02
8.09302405e-03 6.60322412e-02 2.17146476e-02 1.64756385e-01
7.82036621e-02 7.40708952e-03 1.04026589e-01 1.56276119e-01
1.89438907e-01 2.12896502e-01 2.22359639e-01 1.10397939e-01
6.26268128e-01 2.70948458e-01 2.11376743e-01 4.37753043e-01
2.92686083e-01 2.78311532e-01 5.13149772e-01 2.19815297e-01
2.53715996e-01 5.09194435e-01 4.56744044e-01 2.99795330e-01
3.39949498e-02 2.15119911e-01 1.53681354e-01 7.44951119e-03
4.37310351e-01 2.67270601e-01 1.02475274e-01 1.27552193e-01
2.97708126e-02 7.32867604e-01 6.27414398e-01 1.83179228e-01
1.13955373e-01 5.31276621e-01 5.27947680e-01 3.38408064e-02
1.18592363e-01 1.38010611e-01 1.25621722e-01 4.46988762e-01
9.65298219e-02 4.81117386e-01 5.05708301e-01 8.66377640e-02
4.07164549e-01 3.86545451e-01 1.40398278e-01 3.68518682e-01
4.26088040e-01 6.77024848e-02 2.26163948e-01 2.58544768e-01
4.04241908e-01 2.86015995e-01 4.32456271e-01 1.25463503e-01
9.85112307e-02 7.70276493e-02 9.64096460e-02 5.88897387e-01
7.47904779e-02 1.47978260e-02 4.40545002e-02 9.22453421e-02
2.07248739e-01 7.14654998e-02 1.58557269e-02 1.77540412e-01
1.85329660e-01 6.41483922e-01 3.51462818e-01 9.03748043e-02
9.07716033e-02 1.74226386e-01 5.06241863e-01 2.12413220e-01
4.73886919e-01 1.84501968e-01 1.08952266e-01 2.81990474e-01
1.50654900e-01 5.71817967e-02 1.03318209e-01 2.10896925e-01
1.07509299e-01 8.51337952e-02 8.28480205e-02 1.35533391e-01
2.34520344e-01 3.21059749e-01 5.73292029e-02 4.39879479e-02
3.56582486e-01 2.45170550e-02 1.75084581e-02 3.25191233e-01
1.15650402e-01 2.66201050e-01 3.72757273e-01 9.41339402e-02
1.31116897e-01 1.56908618e-01 2.33674801e-01 2.13549886e-01
1.32216151e-01 3.53099365e-01 5.04773331e-02 3.11478398e-02
9.31795064e-02 1.13278918e-01 6.55412595e-03 1.32064075e-01
1.01777811e-01 3.37371902e-01 4.39029220e-01 2.92776546e-01
2.34948584e-01 3.60087821e-01 1.02234410e-01 1.08781434e-01
3.98015325e-01 1.71856842e-01 5.87004024e-01 8.27871479e-02
2.55256134e-01 2.02694350e-01 3.24563288e-01 1.55602275e-01
5.05874811e-01 3.32684694e-01 2.47854351e-01 7.92021148e-02

3.70449407e-01 6.08102310e-02 6.60715230e-01 2.87184681e-01
1.26280821e-02 3.17829228e-01 3.21131933e-01 3.11050952e-04
1.45093690e-02 3.95829289e-01 2.98348309e-01 3.70987344e-01
1.14486115e-01 1.83521262e-01 5.62618095e-04 1.24698917e-01
4.09241407e-01 4.26127187e-01 2.21170114e-02 2.47185594e-01
3.38997330e-01 6.60569821e-02 1.26539173e-01 2.30035251e-01
1.45787841e-01 1.47934074e-01 8.16032069e-02 4.55031384e-01
2.75064762e-01 5.69242343e-02 2.19066204e-01 1.34516106e-01
2.22951800e-02 1.33122707e-01 5.25429147e-01 1.62671554e-01
2.25379418e-01 3.03929902e-01 2.05144225e-01 4.82503071e-01
1.44036629e-01 3.31890628e-01 1.92565210e-01 3.16752990e-01
6.57491269e-02 3.83555997e-01 2.39482060e-01 3.56916914e-02
2.10537280e-01 1.01730931e-01 5.20289255e-02 2.19408143e-01
4.14912017e-02 2.56633825e-01 2.61491881e-02 7.85476795e-02
4.23632335e-01 3.63841726e-01 1.55055947e-01 3.09325595e-02
3.15719498e-02 6.01611017e-01 4.56186181e-01 2.64979981e-01
3.59700448e-02 3.45789544e-01 2.19880123e-01 3.77975263e-01
1.06479284e-01 2.75467801e-01 2.16397779e-02 3.34929262e-01
2.40565927e-01 8.04955831e-02 2.10119442e-01 2.60764297e-01
1.22438934e-01 3.19115427e-01 6.02629945e-02 1.49436441e-01
9.48860412e-02 2.72600655e-01 3.79178522e-01 6.02718304e-01
5.64155301e-01 2.15396365e-01 1.81762193e-02 5.29842629e-02
4.43792787e-01 7.58378640e-02 2.20142815e-01 2.20544198e-02
5.00477242e-01 3.56776591e-01 2.58718641e-01 4.58417710e-01
6.02905980e-01 4.62341936e-01 2.60979312e-02 3.27686589e-01
8.30915133e-02 2.72902745e-01 3.68498373e-01 2.02581643e-02
3.07994335e-01 4.31056285e-01 4.58304101e-01 1.89346763e-01
2.82310000e-01 1.25549881e-01 2.74766520e-01 3.96186480e-01
4.89054288e-01 2.43608774e-01 2.21943606e-01 1.33462417e-01
1.01693552e-01 4.50335956e-01 1.34746632e-01 4.54030650e-01
7.73647536e-02 2.27779215e-01 1.35979903e-01 4.49500886e-02
1.23187372e-01 2.18852169e-01 1.63476233e-01 1.75851351e-01
1.32965931e-02 2.23586859e-01 4.56142431e-01 2.68775966e-01
2.25463391e-01 3.58247231e-02 4.21663667e-01 6.63886959e-01
2.14209055e-01 7.77046033e-01 1.03950348e-02 3.52646242e-01
4.27799444e-01 6.44508633e-02 6.04154396e-01 1.22709665e-01
2.16439451e-01 6.37393217e-01 2.99186880e-01 2.55284127e-01
1.19143981e-01 3.24626546e-01 6.21173598e-02 1.02287094e-01
1.67529835e-01 7.66154942e-01 2.97847040e-01 1.13159054e-01
1.57259787e-01 3.84252515e-01 3.15462715e-01 3.71914069e-01
9.31448033e-02 1.43887902e-02 1.45511128e-01 3.65775961e-01
3.46074662e-01 3.28696635e-01 4.06344415e-01 8.88270460e-02
4.25911911e-01 1.66470400e-01 3.50424846e-01 9.65927560e-02

7.66396102e-02 1.17277752e-01 2.65323566e-01 1.72551150e-01
2.33126930e-01 1.24710752e-02 6.34256310e-01 3.48809257e-01
3.50340032e-01 1.03076865e-01 3.34542440e-01 1.68914367e-02
1.04100503e-01 5.01543219e-01 3.42567619e-01 5.58121755e-01
2.95045807e-01 1.54098697e-01 3.82219948e-01 7.83870588e-02
3.88001443e-02 1.09540410e-01 4.05650393e-02 1.10470027e-01
3.88651711e-01 2.68319028e-01 6.84470876e-02 4.93955931e-02
1.40926762e-01 2.18388204e-01 3.23053076e-02 1.19674201e-01
4.98863138e-01 1.84034311e-01 2.40421402e-01 4.00940755e-01
3.57432833e-01 2.68312269e-01 1.49837566e-01 1.21850974e-01
4.88308863e-01 4.50718743e-01 7.90333112e-02 2.63736920e-01
8.82525655e-02 4.06988366e-01 1.21227944e-01 1.10658358e-01
3.11978116e-01 1.57649690e-01 2.43617662e-02 1.87237460e-01
3.21838281e-01 6.22683787e-01 7.17440790e-02 8.41869929e-03
1.78058272e-01 1.31291106e-01 2.71133804e-01 4.37115402e-01
2.24439073e-01 4.22166850e-02 1.25226635e-01 7.86978305e-02
6.68079876e-02 9.11567138e-02 2.73103491e-01 4.06236910e-01
1.53026217e-01 1.22572888e-02 2.31127792e-01 1.54220455e-01
4.31066663e-01 1.93515762e-03 1.93711852e-01 3.06656715e-01
4.29157867e-01 1.58126072e-01 2.16328058e-01 2.73251233e-01
3.07235614e-01 4.19887915e-01 1.99610078e-01 2.67472536e-02
3.64880492e-01 5.61037334e-01 1.72649783e-02 7.87789533e-02
1.94323599e-01 6.68903833e-03 3.34084718e-01 4.66209716e-01
3.35565116e-01 1.48663471e-01 7.40709055e-02 1.83422817e-01
1.25056105e-01 6.36642833e-02 2.67194786e-01 2.80189099e-01
6.43191982e-01 1.06309959e-01 1.47222363e-01 2.19438372e-01
9.69663638e-02 4.14487326e-02 4.71173453e-01 7.63729762e-03
3.76017330e-01 2.85030267e-01 3.84608534e-01 8.30933279e-02
1.48893346e-01 3.53434172e-01 3.43316450e-01 3.33300776e-01
2.11305560e-02 4.56969909e-01 3.55385469e-02 2.51549447e-01
5.69095368e-01 1.62928386e-01 1.25749327e-01 1.22872690e-01
1.27445697e-01 3.76403479e-01 1.25030988e-02 2.02238050e-02
2.71351946e-01 9.58859674e-02 2.75494140e-01 3.25069996e-01
7.20437852e-01 4.61475446e-01 2.08975299e-01 1.67596745e-02
2.58223536e-01 2.98828087e-01 8.51930033e-02 1.30097786e-03
3.61648567e-01 4.36389952e-03 5.01185462e-02 2.04034376e-01
3.24765942e-01 7.22018729e-02 1.54111729e-01 1.49627666e-01
5.68732912e-02 4.95606737e-01 5.08932815e-01 3.97784764e-02
3.15387026e-02 3.20808932e-01 5.38003217e-02 3.65611260e-01
4.14158990e-01 1.43405237e-01 1.56466244e-01 4.88994167e-01
5.59866568e-01 1.58717981e-01 7.81196457e-02 1.82563559e-01
3.36855474e-01 1.84751776e-02 4.07012536e-01 1.68598581e-01
3.92907059e-01 1.70327402e-01 6.54878926e-02 2.07951055e-02

2.11448364e-02 8.30391438e-02 2.02594608e-01 1.82842043e-01
3.47084516e-01 4.00350871e-02 7.16294583e-02 1.66542586e-01
3.73810495e-02 3.29404250e-01 1.61000440e-01 6.52637398e-02
4.87491258e-01 2.82984638e-01 8.87575226e-02 7.81651360e-02
1.95263640e-02 4.11889148e-01 4.16815281e-01 5.76330694e-01
3.56634202e-02 7.67082990e-02 3.09736729e-02 2.11992802e-02
3.97280163e-01 3.91100140e-02 8.69477233e-02 4.64322411e-01
2.85979788e-01 2.99525693e-01 1.72798649e-01 5.63192662e-01
1.75396026e-02 1.11593229e-01 2.28046437e-01 3.98680942e-01
5.37013262e-03 4.03807233e-01 5.06855160e-01 1.48757294e-01
2.74941090e-01 2.07171204e-01 3.98638194e-01 5.88882216e-01
3.89879589e-01 9.19046762e-02 2.89584103e-01 2.87409914e-01
3.52539873e-01 3.58741651e-01 2.60857938e-01 4.53913007e-01
1.01576279e-01 1.61911533e-01 1.45041270e-01 4.05723667e-01
2.09221738e-01 1.66313358e-01 2.18827748e-01 1.84159747e-01
8.70562033e-02 3.50478890e-02 6.54336703e-01 1.72615144e-01
3.91318770e-01 3.14184397e-01 2.96904548e-01 1.73756096e-01
1.68336143e-01 3.51380068e-01 4.63227096e-01 7.08953810e-03
5.55003291e-01 5.14842810e-03 8.09420036e-02 6.02847630e-01
8.57195960e-01 1.04694910e-01 4.94605740e-02 1.99146348e-02
4.84307571e-01 4.10428150e-01 2.29344035e-01 4.28514599e-01
4.87864753e-01 5.43469543e-01 4.68979905e-02 2.51664756e-01
1.03437719e-02 1.30885918e-01 2.88972640e-02 2.60084307e-01
5.09887594e-01 2.18064417e-01 1.76977995e-01 5.48360512e-01
2.71744120e-01 8.06742998e-02 2.77957200e-01 7.74219857e-02
4.78618583e-02 3.95257102e-01 2.52254295e-01 2.03345035e-01
3.33760931e-01 1.42682288e-01 1.65995915e-01 1.20799931e-01
1.21416175e-01 5.98519286e-02 1.76551240e-01 2.16673383e-01
3.20511693e-01 2.66418219e-01 3.38424338e-01 4.85027850e-01
4.60651779e-02 1.67482361e-01 4.38595610e-02 3.41965124e-01
1.66131597e-01 7.01176900e-02 3.04481271e-01 9.48746079e-02
1.44842354e-01 3.04671900e-01 6.00583590e-01 2.19821176e-01
1.57369136e-01 9.12055979e-02 4.53771664e-01 3.13536854e-01
4.54653878e-01 1.07739848e-01 3.31251348e-01 2.04042222e-01
2.72652509e-01 3.62977879e-01 2.48607566e-01 4.68797474e-01
4.44669945e-02 1.53722487e-01 6.50417960e-02 3.25881987e-01
6.05479760e-02 2.83295287e-01 1.98599073e-01 4.19981020e-01
2.97638624e-01 8.31242410e-02 1.67422334e-01 2.29467364e-02
1.65816183e-02 1.64160487e-01 1.07924812e-01 2.08113603e-01
3.30789945e-01 5.16060968e-01 1.03089048e-01 5.85342239e-01
5.25055598e-02 3.13979215e-01 8.36794452e-02 5.28567135e-01
2.41956110e-01 2.37930773e-01 4.22726093e-01 3.11245418e-01
6.85914829e-01 3.98731667e-04 1.84164836e-02 1.50190279e-01

1.72665510e-02 1.16971712e-01 6.31466248e-02 1.14916110e-02
1.64747305e-01 1.38515074e-02 1.42228319e-02 7.31519983e-02
5.88074147e-01 8.11235902e-02 2.08337242e-01 1.72938745e-01
6.59902964e-01 2.48726041e-01 3.29084561e-01 3.15420243e-01
3.66177883e-02 3.31600405e-02 4.43147889e-01 1.28096113e-01
1.54120669e-01 3.87319532e-01 3.54051999e-01 5.45209183e-01
5.00619976e-03 4.83792150e-02 3.12175315e-01 5.28652103e-01
1.26294352e-02 6.10065087e-01 6.10256645e-02 6.63975693e-02
6.07755209e-01 1.82362343e-02 7.99835950e-02 1.08390318e-01
2.30957500e-01 6.84489464e-02 7.03830589e-01 2.07695564e-01
1.84939444e-01 1.52996098e-01 5.72597119e-02 5.03818726e-01
9.06648910e-02 2.21417703e-01 4.86335236e-02 3.35239027e-01
4.20018169e-02 1.37003930e-01 4.38290279e-02 3.18295514e-01
4.55749185e-01 1.97662270e-01 4.75326333e-03 6.21890120e-01
4.21568316e-01 7.57572100e-02 8.74797260e-02 2.61857405e-01
4.88185344e-01 2.09123564e-01 8.44683669e-02 4.50807187e-01
5.57344082e-01 2.66559732e-01 9.93740181e-02 1.82658879e-01
1.95604637e-01 4.00063398e-02 2.35280159e-01 6.83135619e-03
2.25211488e-01 1.10310244e-01 9.53340095e-02 1.41975023e-01
1.49922860e-01 1.87157060e-01 3.54201875e-01 2.22542874e-01
4.59112648e-01 2.65983248e-01 2.57914611e-01 2.70927336e-01
2.23595569e-01 1.08776679e-01 5.53072071e-02 2.37734057e-01
2.38223249e-01 3.57942679e-01 6.54444936e-02 1.05708043e-01
4.74443507e-01 3.85839643e-03 3.44563149e-01 5.79848819e-02
2.06677067e-01 4.21834759e-01 2.73043693e-01 5.44435048e-03
3.14558641e-01 4.05104882e-01 6.24747161e-01 3.83620775e-01
2.00512214e-02 2.61391931e-01 4.30027383e-01 2.84237411e-01
7.32085652e-02 4.33324910e-01 7.33490094e-01 3.90168155e-01
2.70539512e-01 1.00686315e-01 1.33239181e-01 2.66028059e-01
1.31119045e-01 4.18483844e-01 4.21239320e-01 5.41576851e-01
2.72350592e-01 3.58568256e-01 9.72483883e-02 1.13407253e-01
1.40287019e-01 1.19873156e-01 4.88074590e-01 1.80438577e-01
3.88842693e-01 4.36996026e-01 4.34303567e-02 1.36456457e-01
6.72626136e-02 2.98054593e-01 4.58701993e-01 4.65683555e-01
3.52816520e-01 8.08591688e-02 5.00063059e-01 3.05295501e-01
2.78678829e-01 7.89376324e-01 3.07697686e-01 7.32739257e-02
1.93906857e-03 6.10862198e-02 6.56745095e-02 1.52809199e-01
3.83482815e-01 1.77077489e-01 1.66912359e-01]

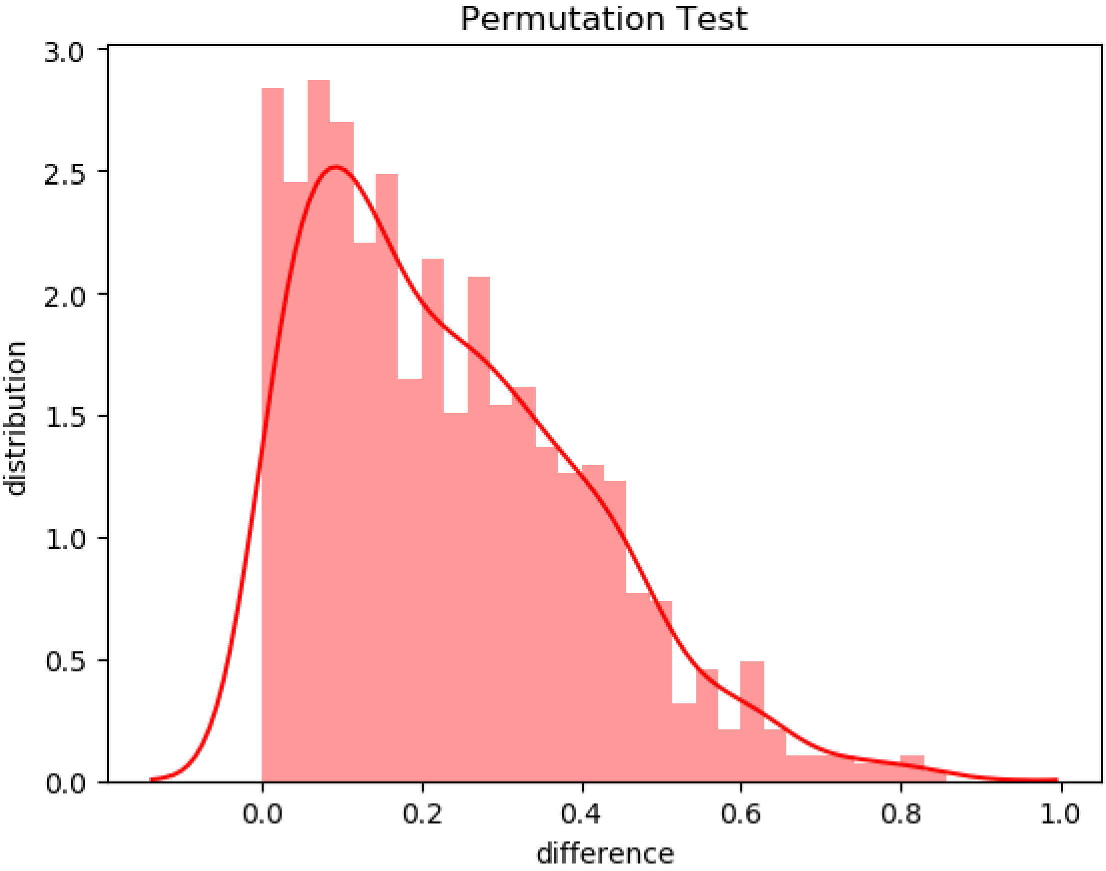

Permutation Test
distribution
difference
3.0
2.5
2.0
1.5
1.0
0.5
0.0
0.0
0.2
0.4
0.6
0.8
1.0
